# Integration Analysis of Three *Omics* Data Using Penalized Regression Methods: An Application to Bladder Cancer

**DOI:** 10.1371/journal.pgen.1005689

**Published:** 2015-12-08

**Authors:** Silvia Pineda, Francisco X. Real, Manolis Kogevinas, Alfredo Carrato, Stephen J. Chanock, Núria Malats, Kristel Van Steen

**Affiliations:** 1 Genetic and Molecular Epidemiology Group, Spanish National Cancer Research Centre (CNIO), Madrid, Spain; 2 Systems and Modeling Unit–BIO3, Montefiore Institute, Liège, Belgium; 3 Epithelial Carcinogenesis Group, Spanish National Cancer Research Centre (CNIO), Madrid, Spain; 4 Departament de Ciències Experimentals i de la Salut, Universitat Pompeu Fabra, Barcelona, Spain; 5 Centre for Research in Environmental Epidemiology (CREAL) and Parc de Salut Mar, Barcelona, Spain; 6 Servicio de Oncología, Hospital Universitario Ramon y Cajal, Madrid, and Servicio de Oncología, Hospital Universitario de Elche, Alicante, Spain; 7 Division of Cancer Epidemiology and Genetics, National Cancer Institute, Department of Health and Human Services, Bethesda, Maryland, United States of America; 8 Systems Biology and Chemical Biology, GIGA-R, Liège, Belgium; The University of Texas M.D. Anderson Cancer Center, UNITED STATES

## Abstract

*Omics* data integration is becoming necessary to investigate the genomic mechanisms involved in complex diseases. During the integration process, many challenges arise such as data heterogeneity, the smaller number of individuals in comparison to the number of parameters, multicollinearity, and interpretation and validation of results due to their complexity and lack of knowledge about biological processes. To overcome some of these issues, innovative statistical approaches are being developed. In this work, we propose a permutation-based method to concomitantly assess significance and correct by multiple testing with the MaxT algorithm. This was applied with penalized regression methods (LASSO and ENET) when exploring relationships between common genetic variants, DNA methylation and gene expression measured in bladder tumor samples. The overall analysis flow consisted of three steps: (1) SNPs/CpGs were selected per each gene probe within 1Mb window upstream and downstream the gene; (2) LASSO and ENET were applied to assess the association between each expression probe and the selected SNPs/CpGs in three multivariable models (SNP, CPG, and Global models, the latter integrating SNPs and CPGs); and (3) the significance of each model was assessed using the permutation-based MaxT method. We identified 48 genes whose expression levels were significantly associated with both SNPs and CPGs. Importantly, 36 (75%) of them were replicated in an independent data set (TCGA) and the performance of the proposed method was checked with a simulation study. We further support our results with a biological interpretation based on an enrichment analysis. The approach we propose allows reducing computational time and is flexible and easy to implement when analyzing several types of *omics* data. Our results highlight the importance of integrating *omics* data by applying appropriate statistical strategies to discover new insights into the complex genetic mechanisms involved in disease conditions.

## Author Summary

At present, it is already possible to generate different type of *omics–*high throughput–data in the same individuals. However, we lack methodology to adequately combine them. Many challenges arise while the amount of data increases and we need to find the way to identify and understand the complex relationships when integrating data. In this regard, new statistical approaches are needed, such as the ones we propose and apply here to integrate three types of *omics* data (genomics, epigenomics, and transcriptomics) generated using bladder cancer tumor samples. These innovative approaches (LASSO and ENET combined with a permutation-based MaxT method) allowed us to find 48 genes whose expression levels were significantly associated with genomics and epigenomics markers. The adequacy of this approach was confirmed by the use of an independent data set from The Cancer Genome Atlas Consortium: 75% of the genes were replicated. Previous sound biological evidences further support the results obtained.

## Introduction

Integrating different *omics* data types, such as genomics, epigenomics and transcriptomics, may provide a new strategy to discover unknown genomic mechanisms involved in complex diseases [1–3]. In cancer, tumor initiation and progression are the consequence of alterations in multiple pathways and biological processes including gene mutations, epigenetic changes, modifications in gene regulation, and environmental influences. In the process to integrate all of this information many challenges arise, among them the high dimensionality of data—since >2 *omics* data sets with millions of measurements are available from the same set of individuals—and the huge heterogeneity of *omics* data due to the different measurement scales [4]. Besides that, the data might be highly correlated, i.e. Single Nucleotide Polymorphisms (SNPs) that are in high linkage disequilibrium (LD) block or DNA CpG sites that belong to the same CpG island, contributing to multicollinearity in the analysis. Another challenge in *omics* data integration regards to the very small number of individuals in comparison to the number of parameters (“n << p”). In addition, interpretation and validation of *omics* derived results require of resources that are still lacking at present. In this rapidly evolving scenario, advanced methodological techniques are continuously emerging, demanding the development of improved data analysis tools [5–7].

Integrative *omics* analysis refers to the combination of at least two different types of *omics* data. Relationships between two sets of *omics* parameters such as the expression quantitative trait loci (eQTL) [2,8,9] or the methylation-QTL (methQTL) [3,10,11], have been recently reported. The approach most commonly used for this type of pairwise analysis has been univariate models (i.e., Spearman/Pearson correlation or linear regression models), assuming that the changes in gene expression levels are only affected by one parameter. Until present, the combination of >2 *omics* data has been less explored.

Towards this end, the previously mentioned challenges are magnified and there is a lack of advanced methodologies to deal with them. Recently, we published an integrative framework as a first approach to integrate genomics, epigenomics, and transcriptomics in individuals with urothelial bladder cancer (UBC) [12]. In that work, we found that some gene expressions were co-regulated by both DNA methylation and genetic variants, both acting together in trans relationships. Therefore, the integration of multiple types of *omics* data by applying multivariable approaches becomes essential to understand the intricacy of the genomic mechanisms behind complex diseases and to overcome the abovementioned challenges.

In this regard, previous developments are Principal Component Analysis (PCA), to reduce data dimensionality, or Canonical Correlation Analysis (CCA) to investigate the overall correlation between two sets of variables. However, these methods are descriptive or exploratory techniques rather than hypothesis-testing tools. While some statistical applications have been developed in an *omics* integrative framework (sparse canonical correlation analysis [13], multiple factor analysis [14], or multivariate partial least square regression [15]), none of them offers the possibility to combine >2 *omics* data together in the same model.

The Least Absolute Shrinkage and Selection Operator (LASSO) proposed by Tibshirani in 1996 [16] and the Elastic Net (ENET) proposed by Hui Zou and Trevor Hastie in 2005 [17] are penalized regression methods that, after appropriate standardization, can model more than one type of *omics* data, face multicollinearity issues, and mitigate the “n << p” problem. More importantly, both methods simultaneously execute variable selection and parameter estimation, thus reducing the computation time, while the traditional methods work on the two problems separately, first selecting the relevant parameters and then computing the estimates. LASSO and ENET have already been applied to GWAS studies [18–20] as well as in the context of integrative studies [21]. One limitation of penalized regression techniques is that the penalty produces biased estimators; consequently, standard errors are not meaningful and cannot provide p-values to assess significance. Here, we propose a permutation-based approach to assess significance and we combine it with a correction for Multiple Testing (MT) using the MaxT algorithm [22]. We apply this permutation-based MaxT method with LASSO and ENET to identify relationships between common genetic variation, DNA methylation, and gene expression, all determined in UBC tumor samples. Specifically, we first built a two *omics* integrative model associating SNPs or CpGs with gene expression levels and, then, we integrated the three *omics* data to assess whether changes in gene expression levels could be confounded/modified by genetic variants and/or DNA methylation.

## Material and Methods

### Penalized regression methods

LASSO and ENET penalized regression methods are applied to high-dimensional problems with a large number of parameters. The penalization produces a shrinkage of the regression coefficients towards zero given a sparse model reducing the irrelevant parameters. Both methods deal with highly correlated variables though in a different way. LASSO tends to select one variable from a group of correlated features whereas ENET selects the whole group of variables, when evidence for their relevance exists. The shrunk estimators introduce a bias while reducing the variance resulting in a better precision and accuracy model and, therefore, increasing its statistical power.

#### Definition of the methods

Consider the standard linear regression model where *y* = (*y*_1_, …*y*_*n*_)^*t*^ is the response variable and *x* = (*x*_1*j*_, …*x*_*nj*_)^*t*^ *j* = 1, …*p* are the standardized predictors, the LASSO solves the *l*_*1*_ penalized regression problem, the Ridge regression [23] solves the *l*_*2*_ penalized regression problem and the ENET is the combination between the *l*_*1*_ and *l*_*2*_ penalized regression problem.

For the LASSO and ENET estimates $\hat{\beta_{0},}\;\hat{\beta }={\left({\hat{\beta }}_{1},\ldots ,{\hat{\beta }}_{p}\right)}^{t}$; $\left(\hat{\beta_{0},}\;\hat{\beta }\right)$ are defined by

$$\left(\;\hat{\beta_{0},}\hat{\beta }\right)=\text{arg}\;min\left\{\sum_{i=1}^{n}{\left(y_{i}-\beta_{0}-\sum_{j=1}^{p}\beta_{j}x_{ij}\right)}^{2}\right\}$$

*with the restrictions*:

$$\sum_{j=1}^{p}|\beta_{j}|\leq t\;(LASSO),$$ (1)

$$\sum_{j=1}^{p}|\beta_{j}|\leq t\;,\sum_{j=1}^{p}\beta_{j}^{2}\leq t\;(ENET).$$ (2)

Here, *t* ≥ 0 is the tuning parameter that controls the amount of shrinkage that is applied to the estimates. For ${\hat{\beta }}_{j}^{0}$ the un-penalized least squares estimate, $t_{0}=\sum |{\hat{\beta }}_{j}^{0}|$. Values of *t* < *t*_0_ will lead to shrinkage towards 0; some coefficients may be exactly equal to 0.

Using the Lagrangian form, this optimization problem is equivalent to (LASSO):

$${\hat{\beta }}_{lasso}\;=\;argmin\left\{\frac{1}{N}\sum_{i=1}^{N}{\left(y_{i}-x_{i}\beta \right)}^{2}+\lambda \;\sum_{j=1}^{p}|\beta_{j}|\right\}$$ (3)

where λ is the penalty parameter related to t. To obtain the optimal penalty, k-fold cross validation (CV) was applied [24] maximizing the penalized log-likelihood function.

(ENET):

$${\hat{\beta }}_{enet}\;=\;argmin\left\{\frac{1}{N}\sum_{i=1}^{N}{\left(y_{i}-x_{i}\beta \right)}^{2}+\lambda_{1}\;\sum_{j=1}^{p}|\beta_{j}|+\lambda_{2}\;\sum_{j=1}^{p}\beta_{j}^{2}\right\},$$ (4)

where λ_1_, λ_2_ are the penalty parameters related to t. In this sense, ENET can be viewed as a penalized least squares method. With *α* = *λ*_2_/(*λ*_1_ + *λ*_2_), solving ${\hat{\beta }}_{enet}$ in Eq (4) is equivalent to the following optimization problem:

$${\hat{\beta }}_{enet}=argmin\left\{\frac{1}{N}\sum_{i=1}^{N}{\left(y_{i}-x_{i}\beta \right)}^{2}+(1-\alpha )\;\sum_{j=1}^{p}\left|\beta_{j}\right|+\alpha \;\sum_{j=1}^{p}\beta_{j}^{2}\right\}$$ (5)

This expression involves a convex combination of the LASSO and ridge penalty. When *α* = 1 the ENET becomes ridge regression and when *α* =0 the ENET becomes LASSO. To obtain the optimal penalty (λ), k-fold CV selecting the best *α* was applied. This value was obtained using a vector of *α* *ϵ*(0.01, 0.99) *by* 0.01.

The LASSO and ENET methods described above were applied to our data with the R package glmnet, that relies on cyclical coordinate descent, computed along a regularization path [25]. To avoid small sample size limitations in variable selection while not introducing an important bias k = 5 was used in the k-fold CV.

These methods are promising in the context of high-throughput data but one of their drawbacks is that they do not provide p-values to assess statistical significance of relationships, nor give a formal assessment of the overall goodness-of-fit. Therefore, a permutation based strategy was adopted to assess significance of discovered relationships combined with a MT correction approach (MaxT algorithm [22]) building upon the statistical concept of deviance. The deviance is used to compare two models and in this case we defined it as

$$Deviance=2[loglik(full_{model})-loglik(null_{model})].$$

Here *loglik* is the loglikelihood function, *full*_*model*_ refers to the model with the parameters selected by LASSO or ENET, and *null*_*model*_ is the model with only the intercept estimated. Thus, the interpretation would be, the higher the deviance the better the model.

### Permutation-based MaxT method

MaxT algorithm of Westfall & Young [22] is a step-down FWER-controlling MT procedure. The method uses the raw p-values or directly the statistics as explained in [26]. Using this approach, the permutation needed to obtain the p-values was combined with the one needed to apply the MaxT algorithm saving computational time. In this work, we used the deviance obtained per each of the permuted LASSO/ENET model to compute the MaxT algorithm and individuals within gene expression measure were permuted, that is the dependent variable in the models. The algorithm is explained in Box 1.

**Box 1. Permutation-based MaxT method**

From the original data, order the deviance obtained per each observed statistics:

$$|D_{s1}|\geq |D_{s2}|\geq |D_{s3}|\geq \cdots \geq |D_{sm}|.$$

For the bth permutation, b = 1…B

1 Permute the n individuals of each of the vectors *Y*_*m*_ = (*y*_1_, … *y*_*n*_)_*m*_
2 Compute the statistics *D*_1*b*,…_ *D*_*mb*_
3 Compute the *U*_*i*,*b*_ = max_*l* = *i*…*m*_|D_*sl*,*b*_|, the successive step-down procedure is: *U*_*m*,*b*_ = |D_*sm*,*b*_|

…

$$U_{2,b}\;=\;\text{max}\left|{\text{D}}_{s2,b},\;{\text{D}}_{s3,b},\;\ldots \;,{\text{D}}_{sm,b}\right|$$

$$U_{1,b}\;=\;\text{max}\left|{\text{D}}_{s1,b},\;{\text{D}}_{s2,b},\;{\text{D}}_{s3,b},\;\ldots \;,{\text{D}}_{sm,b}\right|$$

4 The steps are repeated B times and the adjusted p-values are estimated by:

$$P_{adj,i}=\frac{\#\{b;U_{ib}\geq |D_{si}|\}}{B}\;for\;i=1\;\ldots \;m$$

### Discovery phase: The Spanish Bladder Cancer/EPICURO Study

70 patients with a histologically confirmed UBC were recruited in 2 hospitals during 1997–1998 as part of the pilot phase of the Spanish Bladder Cancer/EPICURO Study. According to established criteria based on tumor stage and grade for UBC, the tumors were classified as low-grade non-muscle invasive, high-grade non-muscle invasive, and muscle invasive. Three sets of *omics* data were obtained using fresh tumor tissue, including common genetic variation (GSE51641), DNA methylation (GSE71666), and gene expression (GSE71576). The three *omics* data overlapped in 27 individuals that are included in this study and comprise 44% low-grade non-muscle invasive tumors, 30% high-grade non-muscle invasive tumors and 26% muscle invasive tumors. S1 Table shows the IDs of the 27 samples used in the following analysis. The local ethics committee of the participating centers approved the study and written informed consent was obtained from all participants at the time of recruitment.

Genotyping of tumor samples was performed using Illumina HumanHap 1M array. A total of 1,047,101 SNPs were determined in 46 individuals and, after the standard quality control and filter the SNPs that were in perfect LD (*r* ^2^ = 1), they resulted in 567,513 SNPs. The application of multivariable models required no missing values, so genotypes were imputed with BEAGLE 3.0 method [27]. CpG methylation data was generated using the Infinium Human Methylation 27 BeadChip Kit. At each CpG site, the methylation levels were measured with M-values using the log2 transformation of the β-values since they are more statistically valid due to a better approximation of the homoscedasticity. The initial number of CpGs in the studied array was 27,578 and after background normalization and QC, a total number of 23,034 CpGs were left for analysis. Gene expression data were obtained from 44 tumor samples using the Affymetrix DNA Microarray Human Gene 1.0 ST Array with 32,321 probes. After the application of QC, it resulted in 20,899 probes determined in 37 individuals. Further details about the preprocessing of the data and the quality control applied can be found elsewhere [12]. The three measures were annotated using the UCSC hg19, NCBI build 37 to make them comparable and homogenize their position in the genome.

### Simulation study

To generate a simulation sample, the association between SNPs and/or CpGs with gene expression was broken and therefore no significant results should be observed. To do that, 10-gene expression probes were randomly selected from our discovery sample showing no correlation structure between the probes and following a multivariate normal distribution. Then, the mean (μ = 8.4) and variance (σ^2^ = 0.4) of all the probes together were obtained. Finally, a simulated set of gene expression probes was generated using the normal distribution obtained and considering the same sample size of the discovery phase (*p* = 20,899 probes and *N* = 27 individuals).

### Replication phase: The Cancer Genome Atlas (TCGA)

UBC tumor data were obtained from The Cancer Genome Atlas (TCGA) consortium (https://tcga-data.nci.nih.gov/tcga/) to replicate our findings. Data was downloaded and processed with the TCGA-Assembler [28]. The study included only individuals with muscle invasive UBC and the tumors were profiled with genome wide 6.0 Affymetrix, RNASeqV2, and HumanMethylation450K Illumina arrays yielding data for 20,502 gene expression probes, 905,422 SNPs, and 350,271 CpGs. The total number of individuals with overlapping data from the three platforms was 238 and they were used in the replication phase of this contribution. S2 Table shows the IDs corresponding to these 238 samples.

### Overall analysis flow

Penalized regression methods LASSO and ENET were applied to the discovery data in combination with the proposed permutation-based MaxT method to select the SNPs and/or CpGs associated with gene expression levels in the following multivariable models:

*SNP model:*

$${Gene\;Expression\;levels}_{i}=\alpha_{1}{SNP}_{1}+\alpha_{2}{SNP}_{2}+\cdots +\alpha_{p}{SNP}_{p};i=1\;\ldots \;m$$

*CPG model:*

$${Gene\;Expression\;levels}_{i}=\gamma_{1}{CPG}_{1}+\gamma_{2}{CPG}_{2}+\cdots +\gamma_{p}{CPG}_{p};i=1\;\ldots \;m$$

*Global model = SNP + CPG model:*

$${Gene\;Expression\;levels}_{i}=\alpha_{1}{SNP}_{1}+\cdots +\alpha_{p}{SNP}_{p}+\gamma_{1}{CPG}_{1}+\cdots +\gamma_{p}{CPG}_{p};i=1\;\ldots \;m$$

**Fig 1:**
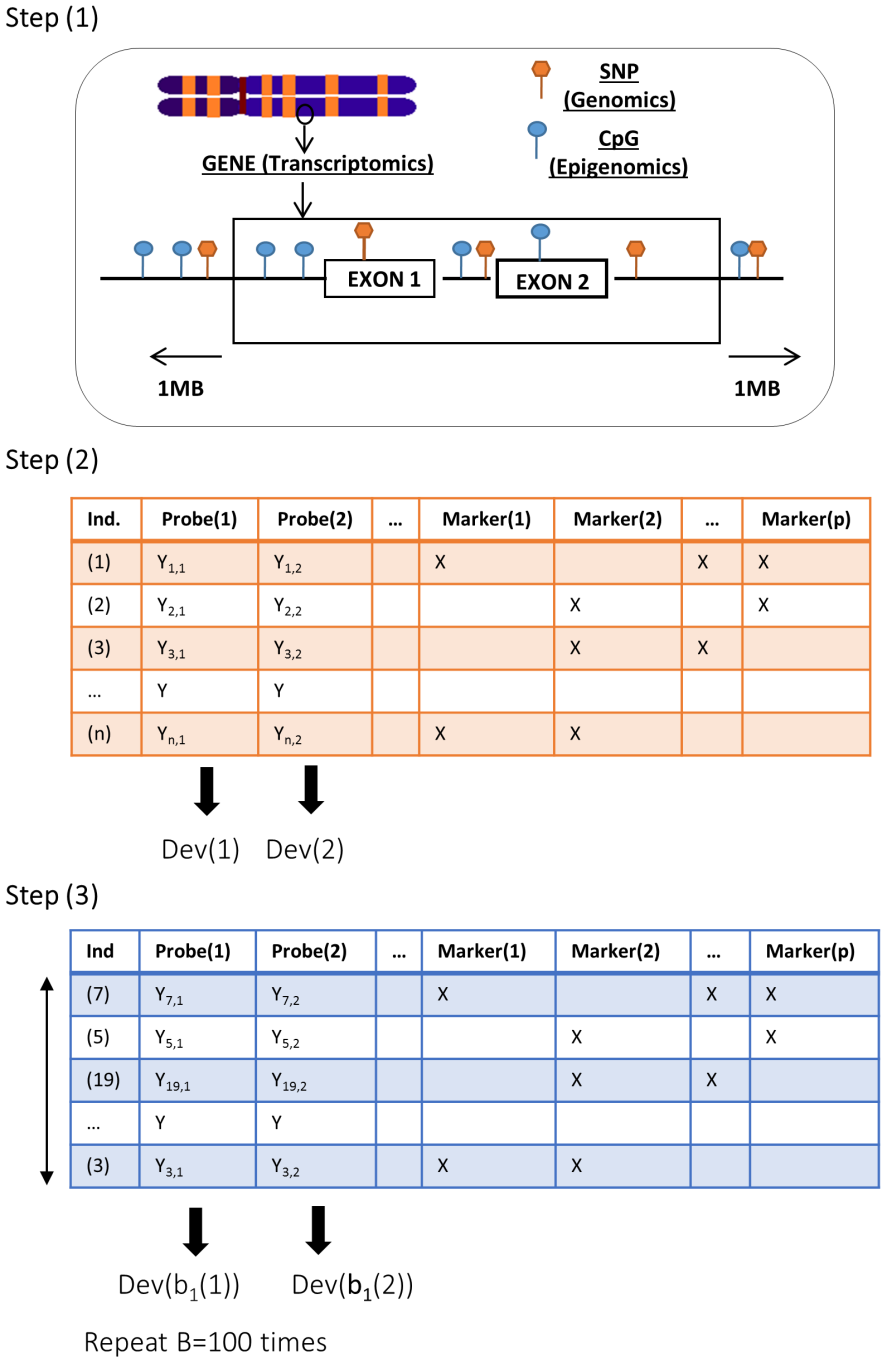
Scenario and workflow of the overall analysis implemented. The integrative framework proposed is based on three steps. Step 1 corresponds to the selection of SNPs and CpGs in 1MB window upstream and downstream from each probe in the gene expression array. Step 2 corresponds to the application of LASSO and ENET to each probe obtaining the deviance per probe. Step 3 corresponds to the permutation-based MaxT method application where gene expression levels within the individuals are permuted B = 100 times obtaining the deviance per probe.

To apply this integrative idea to our set of data the following steps were performed: (1) SNPs and CpGs that were in a 1MB window upstream and downstream were selected from each probe in the gene expression array; (2) LASSO and ENET were applied to each probe and model (SNP, CpG, and Global models) obtaining the deviance per model; and (3), the permutation-based MaxT method was applied to obtain the adjusted p-values (B = 100 permutations and significant adjusted p-value < 0.1). The scenario and workflow is represented in Fig 1.

Subsequently, this analysis flow was applied to the simulated data set using the same criteria. In the replication scenario, we aimed at determining whether the genes that were significant in the discovery phase were also significant in the replication dataset. Therefore, the analysis was restricted to the genes found to be significant in the discovery phase considering all models (SNP, CPG and/or Global) and methods (LASSO and/or ENET). Following the pipeline shown in Fig 1, we focused on the significant genes found in the discovery phase and SNPs and CpGs were selected in 1MB window from the TCGA database, even if the SNPs and CpGs were not the same as those analyzed in the discovery phase. Second, LASSO and/or ENET were conducted to SNP, CPG, and/or Global models. Finally, the permutation-based MaxT method was applied to obtain significance and correct for multiple testing. The replication analysis was performed with the same software and criteria as in the discovery analysis.

### Gene enrichment analysis

To provide a biological interpretation to the results, the entire list of the significant genes identified in the discovery phase by both LASSO and ENET, and by the three models, was used to perform a gene enrichment analysis with the bioinformatics tool DAVID [29,30]. The functional annotation clustering analysis module offered by DAVID was used. The gene term annotation is based on 14 annotation categories (Gene Ontology (GO), Biological process, GO Molecular Function, GO Cellular Component, KEGG Pathways, BioCarta Pathways, Swiss-Prot Keywords, BBID Pathways, SMART Domains, NIH Genetics Association DB, UniProt Sequence Features, COG/KOG Ontology, NCBI OMIM, InterPro Domains, and PIR Super-Family Names) collected in the DAVID tool knowledgebase (https://david.ncifcrf.gov/knowledgebase/DAVID_knowledgebase.html). The method identifies related genes by measuring the similarity of their global annotation profiles. So, the “grouping term” is based on the idea that two genes that have similar annotation profiles are functionally related. Each group term provides an enrichment score (ES) that indicates biological significance when ≥1.3 (equivalent to non-log scale 0.05). DAVID also provides a p-value to examine the significance of gene-term enrichment, which is corrected by Benjamini MT [31].

## Results

### Discovery phase

**Fig 2:**
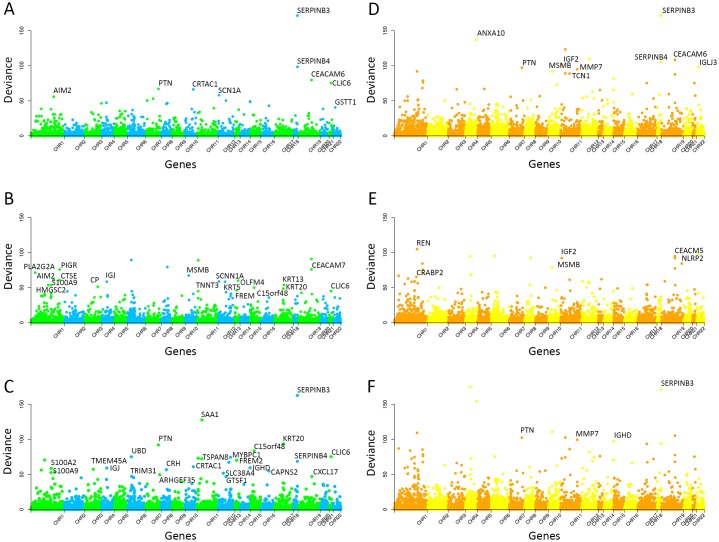
Deviance across the genome when applying LASSO and ENET to select SNPs, CpGs or both (Global model). The dots in the figure indicate the deviance of each gene located in the corresponding position in the genome. There are a total of 20,899 gene expression probes measured. Significant genes after applying the permutation-based MaxT method are tagged. The figures represent the deviance per gene expression probe using LASSO for the SNP model (A), the CpG model (B) and the Global model (C) and using ENET for the SNP model (D), the CpG model (E) and the Global model (F).

LASSO and ENET were applied to 20,899 gene expression probes in each of the three models. Under the conditions mentioned above, LASSO yielded 9 genes with a significant signal in the SNP model, 19 in the CpG model, and 23 in the Global model. Table 1 shows the significant genes, mapped to each probe, with its deviance and p-value. Fig 2A–2C display all the probes analyzed with their deviances represented across the genome. Detailed information about the SNPs and/or CpGs mapped to these genes is provided as Supplementary Material (S1–S6 Excel). ENET identified a lower number of significant genes: 11 in the SNP model, 6 in the CpG model, and 4 in the Global model. These results are shown in Table 2 and Fig 2D–2F. When the MT correction threshold was relaxed, ENET provided additional significant genes.

**Table 1:** Statistically significant genes associated with SNPs and/or CpGs selected by LASSO&Permuted based maxT algorithm.

Gene Name	Chromosome	Model	Deviance	p-value^1^
*AIM2*	1	SNPs	55.8	0.1
		CpGs	61.5	0.06
*PLA2G2A*	1	CpGs	71.4	0.01
*S100A9*	1	CpGs	53.7	0.03
		SNPs + CpGs	52.4	0.08
*HMGCS2*	1	CpGs	53.3	0.02
*PIGR*	1	CpGs	75.8	< 0.01
*CTSE*	1	CpGs	60.7	0.06
*S100A2*	1	SNPs + CpGs	58.7	0.04
*CP*	3	CpGs	51.1	0.02
*TMEM45A*	3	SNPs + CpGs	57.3	0.08
*IGJ*	4	CpGs	58.4	0.03
		SNPs + CpGs	59.0	0.09
*UBD*	6	SNPs + CpGs	75.0	0.07
*TRIM31*	6	SNPs + CpGs	47.1	0.1
*PTN*	7	SNPs	67.0	0.08
		SNPs + CpGs	92.0	< 0.01
*ARHGEF35*	7	SNPs + CpGs	49.6	0.09
*CRH*	8	SNPs + CpGs	56.7	0.1
*CRTAC1*	10	SNPs	66.2	0.03
*MSMB*	10	CpGs	67.3	0.06
*CRTAC1*	10	SNPs	60.9	0.1
		SNPs + CpGs		
*TNNT3*	11	CpGs	44.9	0.09
*SAA1*	11	SNPs + CpGs	127.8	0.04
*SCCN1A*	12	SNPs	57.9	0.08
		CpGs	58.8	0.03
*KRT5*	12	CpGs	58.2	0.03
*TSPAN8*	12	SNPs + CpGs	67.2	0.05
*MYBPC1*	12	SNPs + CpGs	74.5	0.08
*SLC38A4*	12	SNPs + CpGs	51.7	0.08
*GTSF1*	12	SNPs + CpGs	46.7	0.1
*OLFM4*	13	CpGs	60.0	0.06
*FREM2*	13	CpGs	46.0	0.06
		SNPs + CpGs	70.2	0.06
*IGHD*	14	SNPs + CpGs	59.4	0.1
*C15orf48*	15	CpGs	49.9	0.02
		SNPs + CpGs	83.7	0.05
*CAPNS2*	16	SNPs + CpGs	54.9	0.07
*KRT20*	17	CpGs	48.4	0.05
		SNPs + CpGs	93.7	< 0.01
*KRT13*	17	CpGs	53.6	0.02
*SERPINB4*	18	SNPs	98.4	< 0.01
		SNPs + CpGs	68.5	0.03
*SERPINB3*	18	SNPs	171.6	< 0.01
		SNPs + CpGs	162.7	< 0.01
*CEACAM7*	19	CpGs	76.0	< 0.01
*CEACAM6*	19	SNPs	79.6	0.01
*CXCL17*	19	SNPs + CpGs	46.8	0.1
*CLIC6*	21	SNPs	75.3	0.01
		CpGs	45.1	0.09
		SNPs + CpGs	75.3	0.07
*GSTT1*	22	SNPs	40.4	0.07

^1^The p-value was obtained after applying the permuted based–maxT algorithm and was therefore corrected for MT.

**Table 2:** Statistically significant genes associated with SNPs and/or CpGs selected by ENET&Permuted based maxT algorithm.

Gene Name	Chromosome	Model	Deviance	p-value^1^
*REN*	1	CPG	84.3	0.03
*CRABP2*	1	CPG	65.2	0.09
*ANXA10*	4	SNP	137.0	0.01
*PTN*	7	SNP	97.2	0.07
		SNP +CPG	102.5	0.09
*MSMB*	10	SNP	91.8	0.07
		CPG	78.9	0.06
*MMP7*	11	SNP	94.8	0.06
*TCN1*	11	SNP	88.9	0.07
*IGF2*	11	SNP	101.6	0.05
		CPG	92.1	0.04
*MMP7*	11	SNP + CPG	99.4	0.08
*GTSF1*	12	SNP	109.6	0.05
*IGHD*	14	SNP + CPG	97.5	0.1
*SERPINB4*	18	SNP	105.2	0.04
*SERPINB3*	18	SNP	171.6	0.02
		SNP + CPG	171.3	0.01
*CEACAM6*	19	SNP	108.4	0.03
*NRLP2*	19	CPG	84.2	0.04
*CEACAM5*	19	CPG	92.1	0.06
*IGLJ3*	22	SNP	97.7	0.05

^1^The p-value was obtained after applying the permuted based–maxT algorithm and corrected by MT.

**Fig 3:**
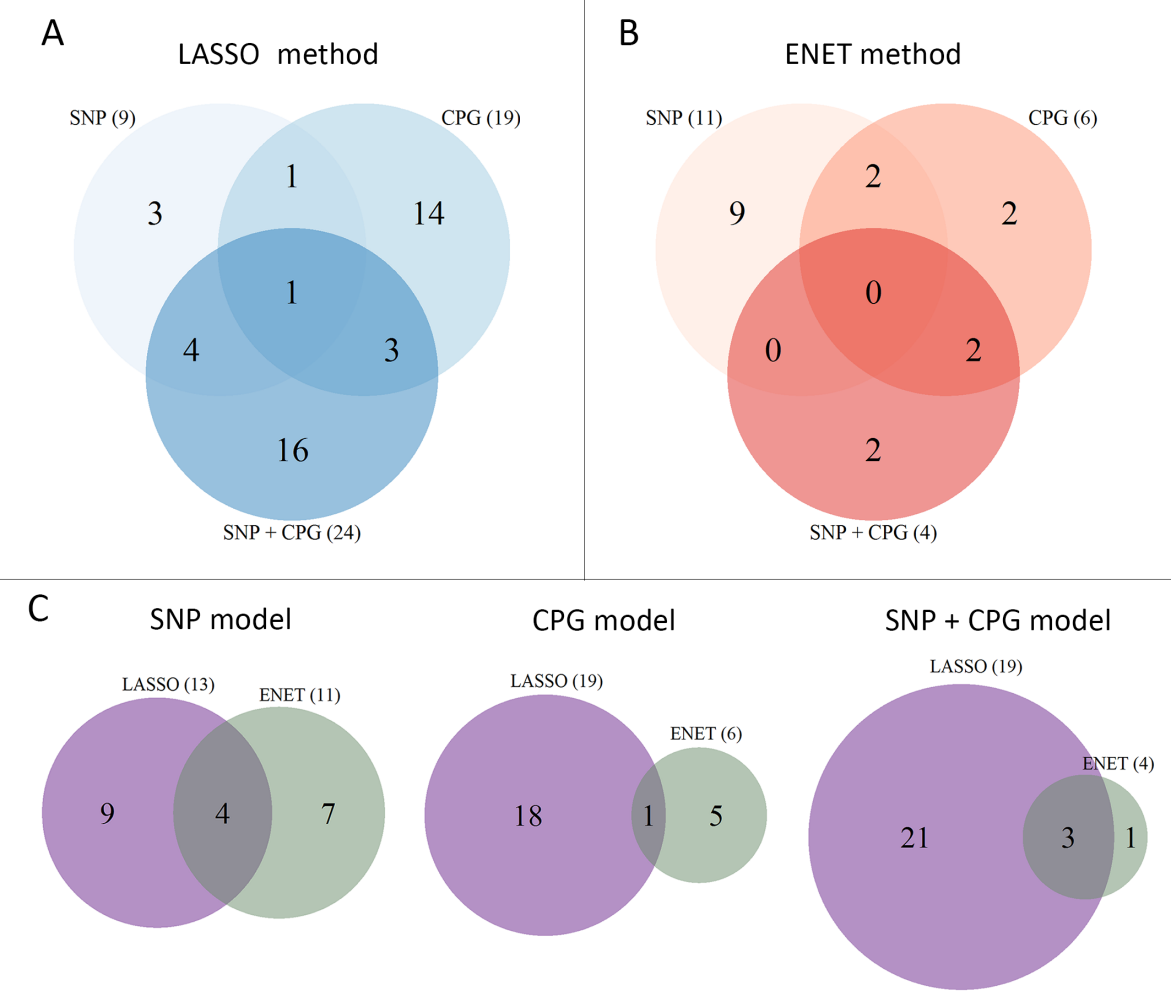
Venn diagrams showing the overlap between the significant genes compared by the two methods (LASSO and ENET) and models (SNPs, CpGs and Global). (A) Number of significant genes using the LASSO method for the three models (SNP, CPG, and Global); (B) number of significant genes using the ENET method for the three models (SNP, CPG and Global); and (C) number of significant genes per model comparing the two methods (LASSO and ENET).

Some genes overlapped among methods and models: *CLIC6* was identified by the three LASSO models; *AIM2* and *SCNN1A* came out in the SNP and CpG models; *PTN*, *CRTAC1*, *SERPINB3* and *SERPINB4* were identified in the SNP and Global models; and *S100A9*, *IGJ*, *FREM2*, *C15orf48* and *KRT20* emerged in the CpG and Global models. Interestingly, 15 genes showed significance in the Global model when combining 3 *omics* data while they were not detected when analyzing only 2 types of *omics* data. The overlap of genes identified by the ENET model was lower: *MSMB* and *IGF2* were identified by the SNP and CpG models, and *PTN* and *SERPINB3* were selected by the SNP and the Global models. When comparing the methods, an overlap between LASSO and ENET was found for four (*PTN*, *SERPINB3*, *SERPINB4* and *CEACAM6*), one (*MSMB*), and three (*SERPINB3*, *PTN* and *IGHD*) significant genes in the SNP, CpG, and Global models, respectively. These results are displayed in Fig 3 using Venn diagrams. In the simulation study, as expected, no gene was significantly associated with any of the two methods and the three models. An example of the deviances of each gene for the SNP 303 model and LASSO method is shown in S1 Fig.

### Replication phase

The replication study was restricted to those genes (n = 48) that showed significant results in the discovery phase and we applied the same models, methods, and criteria of analysis to the TCGA data. Overall, we were able to replicate 75% of the results: 36 out of the 48 genes yielded a significant association at least in one of the models considered. Regarding the LASSO models, we replicated 3/9 genes from the SNP models, 17/19 genes from the CPG models, and 19/23 genes from the Global models (Table 3). Regarding ENET, we replicated 3/10 genes from the SNP model, 3/6 genes from the CPG model, and 3/3 genes from the Global model (Table 4).

**Table 3:** Significant genes obtained by LASSO&Permuted based maxT algorithm for the three models (SNP, CPG, and Global) in the original dataset (EPICURO Study) and the replication dataset (TCGA).

	Original Data (EPICURO)												Validation Data (TCGA)				
	Gene	probeset	Chr	Start	end	Dev	p-value	SNPs (N)	CpGs (N)	Dev	p-value^1^	SNPs (N)	CpGs (N)	SNPs (overlap)	SNPs (rep)	CpGs (overlap)	CpGs (rep)
SNP model	*SERPINB3*	8023696	18	61322433	61329197	171.6	<0.01	29		0	1	0		3	0		
	*SERPINB4*	8023688	18	61304495	61311502	98.4	<0.01	15		0	1	0		2	0		
	*CEACAM6*	8029098	19	42259398	42276113	79.6	0.01	10		0	1	0		0	0		
	*CLIC6*	8068383	21	36041688	36090519	75.3	0.01	30		3.5E-08	0.9	1		14	0		
	***CRTAC1***	**7935535**	**10**	**99624758**	**99790585**	**66.2**	**0.03**	**18**		**2.4E+09**	**0.001**	**12**		**4**	**1 (LD)**		
	***GSTT1***	**8074980**	**22**	**24376141**	**24384284**	**40.4**	**0.07**	**16**		**8.3E+07**	**<0.001**	**34**		**4**	**1 (LD)**		
	*PTN*	8143144	7	136912092	137028546	67.0	0.08	9		0	1	0		1	0		
	*SCNN1A*	7960529	12	6456011	6486523	57.9	0.08	26		0	1	0		8	0		
	***AIM2***	**7921434**	**1**	**159032275**	**159046647**	**55.8**	**0.1**	**6**		**5.7E+05**	**0.03**	**1**		**2**	**0**		
CPG model	***CEACAM7***	**8037053**	**19**	**42177235**	**42192096**	**76.0**	**< 0.01**		**19**	**1.5E+07**	**< 0.001**		**2**			**17**	**0**
	***PIGR***	**7923929**	**1**	**207101869**	**207119811**	**75.8**	**< 0.01**		**21**	**6.4E+08**	**0.001**		**19**			**18**	**1**
	***PLA2G2A***	**7913216**	**1**	**20301925**	**20306932**	**71.4**	**0.01**		**10**	**5.2E+09**	**< 0.001**		**57**			**9**	**0**
	***CP***	**8091385**	**3**	**148890292**	**148939832**	**51.1**	**0.02**		**3**	**1.6E+09**	**< 0.001**		**24**			**1**	**0**
	*HMGCS2*	7919055	1	120290620	120311555	53.3	0.02		8	0	1		**0**			8	-
	***KRT5***	**7963427**	**12**	**52908361**	**52914243**	**58.2**	**0.02**		**25**	**3.6E+12**	**< 0.001**		**112**			**24**	**5**
	***C15orf48***	**7983478**	**15**	**45722763**	**45725645**	**49.9**	**0.02**		**7**	**1.5E+08**	**< 0.001**		**23**			**5**	**0**
	***KRT13***	**8015323**	**17**	**39657233**	**39661865**	**53.6**	**0.02**		**8**	**8.2E+11**	**< 0.001**		**5**			**6**	**0**
	***IGJ***	**8100827**	**4**	**71521259**	**71532348**	**58.4**	**0.03**		**2**	**4.2E+08**	**< 0.001**		**19**			**2**	**0**
	***SCNN1A***	**7960529**	**12**	**6456011**	**6486523**	**58.8**	**0.03**		**29**	**2.1E+09**	**< 0.001**		**12**			**27**	**0**
	***S100A9***	**7905571**	**1**	**153330330**	**153333502**	**53.7**	**0.04**		**11**	**5.0E+11**	**< 0.001**		**33**			**9**	**1**
	***KRT20***	**8015124**	**17**	**39032141**	**39041495**	**48.4**	**0.05**		**3**	**5.9E+09**	**< 0.001**		**45**			**3**	**0**
	***CTSE***	**7909164**	**1**	**206317459**	**206332103**	**60.7**	**0.06**		**12**	**3.4E+09**	**< 0.001**		**36**			**12**	**1**
	***AIM2***	**7921434**	**1**	**159032275**	**159046647**	**61.5**	**0.06**		**8**	**4.7E+07**	**0.002**		**27**			**4**	**0**
	***OLFM4***	**7969288**	**13**	**53602972**	**53626186**	**60.0**	**0.06**		**10**	**1.6E+10**	**< 0.001**		**47**			**9**	**6**
	*MSMB*	7927529	10	51549553	51562590	67.3	0.06		7	0	1		**0**			6	0
	***FREM2***	**7968678**	**13**	**39261173**	**39461265**	**46.0**	**0.08**		**2**	**4.4E+07**	**< 0.001**		**13**			**1**	**0**
	***CLIC6***	**8068383**	**21**	**36041688**	**36090519**	**45.1**	**0.09**		**4**	**1.2E+08**	**< 0.001**		**19**			**4**	**0**
	***TNNT3***	**7937749**	**11**	**1940799**	**1959935**	**44.9**	**0.09**		**26**	**5.2E+08**	**< 0.001**		**72**			**22**	**0**
Global model	***SERPINB3***	**7920285**	**18**	**61322433**	**61329197**	**162.7**	**<0.01**	**15**	**0**	**3.0E+09**	**<0.001**	**6**	**4**	**1**	**0**	**0**	**0**
	***KRT20***	**7905571**	**17**	**39032141**	**39041495**	**93.7**	**<0.01**	**19**	**7**	**5.7E+09**	**<0.001**	**8**	**38**	**0**	**0**	**0**	**0**
	***PTN***	**7935535**	**7**	**136912092**	**137028546**	**92.0**	**<0.01**	**12**	**0**	**2.6E+08**	**<0.001**	**0**	**1**	**0**	**0**	**0**	**0**
	***SERPINB4***	**7938758**	**18**	**61304495**	**61311502**	**68.6**	**0.03**	**4**	**0**	**7.9E+08**	**<0.001**	**27**	**11**	**0**	**0**	**0**	**0**
	*SAA1*	7962559	11	18287808	18291521	127.8	0.04	20	1	7.9E+08	0.6	0	1	5	1	0	0
	***S100A2***	**7957966**	**1**	**153533587**	**153538306**	**58.7**	**0.04**	**20**	**7**	**1.0E+11**	**<0.001**	**1**	**5**	**5**	**0**	**3**	**0**
	***C15orf48***	**7964927**	**15**	**45722763**	**45725645**	**83.7**	**0.05**	**19**	**6**	**1.7E-07**	**<0.001**	**1**	**6**	**0**	**0**	**0**	**0**
	*TSPAN8*	7963817	12	71518877	71551779	67.2	0.05	8	1	9.9E+05	0.02	1	0	3	0	1	0
	***FREM2***	**7968678**	**13**	**39261173**	**39461265**	**70.2**	**0.06**	**14**	**2**	**2.9E+07**	**<0.001**	**3**	**10**	**3**	**0**	**1**	**0**
	***CLIC6***	**7983478**	**21**	**36041688**	**36090519**	**75.3**	**0.07**	**25**	**2**	**1.4E+08**	**<0.001**	**21**	**15**	**0**	**1 (LD)**	**0**	**0**
	***UBD***	**7995712**	**6**	**29523390**	**29527702**	**75.0**	**0.07**	**6**	**5**	**8.8E+08**	**<0.001**	**0**	**25**	**0**	**0**	**0**	**0**
	***CAPNS2***	**7981724**	**16**	**55600584**	**55601592**	**54.9**	**0.07**	**8**	**1**	**5.8E+07**	**<0.001**	**10**	**12**	**0**	**0**	**0**	**0**
	*MYBPC1*	8023688	12	101988747	102079657	74.5	0.08	23	3	9.9E-08	1	0	1	2	0	2	0
	***TMEM45A***	**8037197**	**3**	**100211463**	**100296285**	**57.3**	**0.08**	**12**	**0**	**1.6E+08**	**0.001**	**11**	**19**	**0**	**1 (LD)**	**0**	**0**
	***S100A9***	**8015124**	**1**	**153330330**	**153333502**	**52.5**	**0.08**	**6**	**4**	**4.9E+11**	**<0.001**	**15**	**24**	**0**	**1 (LD)**	**4**	**1**
	***SLC38A4***	**8023696**	**12**	**47158544**	**47219780**	**51.7**	**0.08**	**15**	**1**	**1.6E+08**	**0.001**	**8**	**15**	**6**	**0**	**1**	**0**
	*IGJ*	8068383	4	71521259	71532348	59.0	0.09	3	2	3.3E+08	0.003	1	3	0	0	0	0
	***ARHGEF5***	**8081288**	**7**	**143883177**	**143892791**	**49.6**	**0.09**	**8**	**0**	**1.2E+07**	**<0.001**	**11**	**8**	**0**	**1 (LD)**	**0**	**0**
	***CRTAC1***	**8100827**	**10**	**99624758**	**99790585**	**60.9**	**0.1**	**7**	**5**	**3.8E+09**	**<0.001**	**7**	**9**	**1**	**0**	**3**	**1**
	*IGHD*	8136981	14	106303102	106312014	59.4	0.1	7	1	-	-	-	-	-	-	-	-
	***CRH***	**8151092**	**8**	**67088612**	**67090846**	**56.7**	**0.1**	**3**	**0**	**9.4E+08**	**<0.001**	**7**	**10**	**0**	**0**	**0**	**0**
	***TRIM31***	**8178330**	**6**	**30070674**	**30080867**	**47.1**	**0.1**	**23**	**4**	**5.8E+08**	**<0.001**	**0**	**43**	**0**	**0**	**0**	**0**
	***CXCL17***	**8143144**	**19**	**42932696**	**42947136**	**46.8**	**0.1**	**3**	**5**	**7.4E+08**	**<0.001**	**8**	**11**	**0**	**0**	**0**	**0**
	***GTSF1***	**8124650**	**12**	**54849737**	**54867386**	**46.7**	**0.1**	**2**	**1**	**2.1E+07**	**<0.001**	**18**	**46**	**2**	**0**	**1**	**1**

^1^Bonferroni correction for the p-value were: 0.005 (SNP model), 0.003 (CPG model) and 0.002 (Global model); SNPs (N) and CpGs (N) are the number of SNPs and CpGs that were selected by LASSO per each gene expression probe in EPICURO data with the Illumina HumanHap 1M array and the Methylation 27k array; SNPs (overlap) and CpGs (overlap) are the number of SNPs and CpGs that were present in the TCGA data with the Genome wide 6.0 Affymetrix and the Methylation 450k array; and the SNPs (rep) and CpGs (rep) are the ones selected by LASSO in the TCGA data in common with the EPICURO data. The gene with “no p-value” is a gene that was not present in the RNASeqV2 in TCGA data.

**Table 4:** Significant genes obtained by ENET&Permuted based maxT algorithm for the three models (SNP, CPG, and Global) in the original dataset (EPICURO Study) and the replication dataset (TCGA).

	Original Data (EPICURO)												Validation Data (TCGA)				
	Gene	probeset	Chr	Start	end	Dev	p-value	SNPs (N)	CpGs (N)	Dev	p-value^1^	SNPs (N)	CpGs (N)	SNPs (overlap)	SNPs (rep)	CpGs (overlap)	CpGs (rep)
SNP model	**ANXA10**	**8098246**	**4**	**169013707**	**169108891**	**137.0**	**0.01**	**17**		**1.4E+08**	**<0.001**	**13**		**7**	**1**		
	SERPINB3	8023696	18	61322433	61329197	171.6	0.02	30		1.4E+09	0.08	32		3	0		
	CEACAM6	8029098	19	42259398	42276113	108.4	0.03	28		1.4E+09	0.04	4		5	0		
	SERPINB4	8023688	18	61304495	61311502	105.2	0.04	31		1.1E+08	0.07	10		8	1 (LD)		
	GTSF1	7963817	12	54849737	54867386	109.6	0.05	19		1.6E+06	0.08	7		9	2 (LD)		
	**IGF2**	**7937772**	**11**	**2150348**	**2170833**	**101.6**	**0.05**	**56**		**3.9E+12**	**0.002**	**31**		**12**	**0**		
	IGLJ3	7981730	22	23247030	23247205	97.7	0.05	183		-	-	-		-	-		
	**MMP7**	**7951217**	**11**	**102391240**	**102401478**	**94.8**	**0.06**	**19**		**2.8E+08**	**0.004**	**10**		**6**	**1 (LD)**		
	PTN	8143144	7	136912092	137028546	97.2	0.07	24		0	1	0		10	0		
	MSMB	7927529	10	51549553	51562590	91.8	0.07	78		0	1	0		0	0		
	TCN1	7948444	11	59620281	59634041	88.9	0.07	122		0	1	0		0	0		
CPG model	*REN*	7923608	1	204123944	204135465	84.3	0.03		22	0	1		1			22	0
	***IGF2***	**7937772**	**11**	**2150348**	**2170833**	**92.1**	**0.04**		**15**	**8.1E+12**	**<0.001**		**609**			**12**	**7**
	***NLRP2***	**8031398**	**19**	**55476652**	**55512508**	**84.2**	**0.04**		**34**	**8.0E+08**	**< 0.001**		**10**			**28**	**2**
	*CEACAM5*	8029086	19	42212530	42234436	92.1	0.06		26	9.3E+08	0.009		1			23	0
	*MSMB*	7927529	10	51549553	51562590	78.9	0.06		9	6.2E+07	0.3		36			7	1
	***CRABP2***	**7921099**	**1**	**156669410**	**156675375**	**65.2**	**0.09**		**39**	**1.1E+10**	**<0.001**		**132**			**35**	**11**
Global model	***SERPINB3***	**7920285**	**18**	**61322433**	**61329197**	**171.3**	**0.01**	**27**	**1**	**5.3E+09**	**<0.001**	**37**	**15**	**0**	**0**	**1**	**1**
	***MMP7***	**7951217**	**11**	**102391240**	**102401478**	**99.4**	**0.08**	**62**	**18**	**2.3E+08**	**0.003**	**5**	**2**	**0**	**0**	**0**	**0**
	***PTN***	**8143144**	**7**	**136912092**	**137028546**	**102.5**	**0.09**	**20**	**0**	**6.1E+08**	**<0.001**	**16**	**15**	**0**	**0**	**0**	**0**
	*IGHD*	7981724	14	106303102	106312014	97.5	0.1	35	6	-	-	-	-	-	-	-	-

^1^Bonferroni correction for the p-values is: 0.008 (CPG model) and 0.01 (Global model); SNPs (N) and CpGs (N) are the number of SNPs and CpGs that were selected by ENET per each gene expression probe in EPICURO data with the Illumina HumanHap 1M array and the Methylation 27k array; SNPs (overlap) and CpGs (overlap) are the number of SNPs and CpGs that were present in the TCGA data with the Genome wide 6.0 Affymetrix and the Methylation 450k array and the SNPs (rep) and CpGs (rep) are the ones selected by ENET in the TCGA data in common with the EPICURO data. The gene with no p-value is a gene that was not present in the RNASeqV2 in TCGA data.

### Gene enrichment analysis

Using DAVID, 46 out of 48 genes showing significant signals in the discovery phase were annotated from 14 public categories. After enrichment analysis, 7 clusters with an ES ≥1.3 were found (S3 Table). The cluster with the highest ES (3.5) regarded to the terms “extracellular region, secreted, and signal peptide” grouping the genes *OLFM4*, *CRTAC1*, *MSMB*, *IGJ*, *MMP7*, *IGF2*, *PIGR*, *TCN1*, *CXCL17*, *S100A9*, *SAA1*, *IGHD*, *CRH*, *CTSE*, *FREM2*, *PLA2G2A*, *CEACAM7*, *CEACAM6*, *CEACAM5*, *REN*, *PTN*, *CP*.

The rest of the clusters with an ES ≥1.3 were not significant after MT correction. Cluster 5 (ES = 1.4) contains 3 genes coding for keratins (*KRT5*, *KRT13*, *KRT20*), cytoskeletal components that are regulated during urothelial differentiation, whose expression is altered in UBC, that have been proposed as markers for the molecular taxonomy of UBC [32]. In addition, cluster 7 “EF hand and calcium ion binding” (ES = 1.3) contains multiple genes shown to play an important role in cancer (*S100A9*, *S100A2*, *CAPNS2*, *ANXA10*, *CRTAC1*, *FREM2*, *MMP7*, *PLA2G2A*), including two members of the S100A family of proteins.

## Discussion

Integration analysis is an emerging area in the field of *omics* data analysis to find new biological insights into complex traits [33]. In this regard, our pathophysiological understanding of cancer could be improved by using innovative approaches based on *omics* data to identify hidden mechanisms in which multiple factors are involved. We previously analyzed the set of *omics* data used here following a multi-stage approach by proposing an *omics* integration analysis framework. The results of this previous work highlighted relevant *omics* trans-acting relationships in UBC [12]. Here, we propose an *omics* integrative analysis pipeline using LASSO and ENET, and focus on cis-acting relationships that appear to have a predominant role in the regulation of gene expression [34]. The three *omics* data are combined in a large input matrix and then a permutationbased MaxT method is adapted to assess the significant models while correcting for MT.

In comparison with classical approaches [6,7], our strategy has several advantages, including the possibility of working with a large number of parameters, even if the sample size is small, dealing with more than one set of heterogeneous data with highly-correlated variables, and providing MT corrected p-values to assess the models’ goodness of fit. Furthermore, the results are easily interpretable due to the dimensionality reduction during the variable selection process.

The expression of 48 genes was found to be significantly associated with SNPs and CpGs in UBC, pointing to new mechanisms in an intricate scenario where common genetic variants and DNA methylation regulate gene expression in cis-acting (1MB) relationships. Some of the genes were identified by the three models and by the two methods, likely underscoring the existence of true relationships.

**Fig 4:**
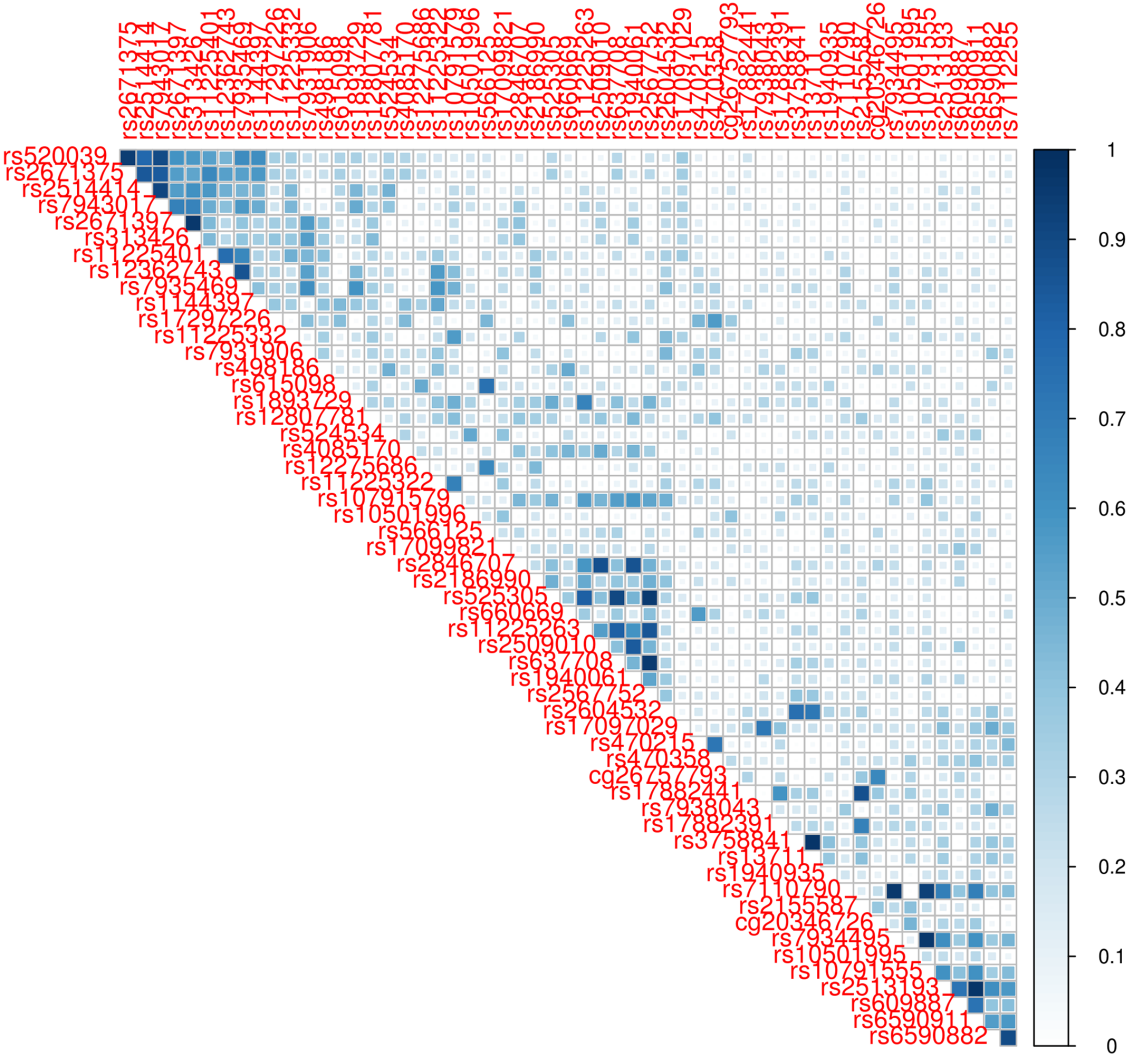
Example of a correlation plot for *MMP7* detected by the Global model using ENET but not using LASSO. The bar color represents the levels of correlation from 0 (no correlation) to 1 (perfect correlation) between SNPs and CpGs that were selected for the *MMP7* models. Three nets of correlated variables are the ones responsible that the gene is only selected by ENET and not by LASSO.

The application of LASSO and ENET as part of the aforementioned integrative analysis framework led to different results. This is not surprising, mainly for two reasons: (1) the *α* parameter (Eq 5) used by LASSO is always equal to 1 while ENET uses *α* < 1. This gives a smaller penalization and therefore more variables with *β* ≠ 0 were foreseen using ENET; and (2) the fact that SNPs and CpGs may be correlated, mainly when they are closely positioned in the genome, leads LASSO to select one from the set of parameters that are highly correlated while ENET forms groups of nets with these variables. In our analysis, only 4/24 (SNP model), 1/25 (CpG model) and 3/28 (Global model) genes were shared by both methods. The genes detected only by LASSO showed large deviances and borderline p-values with ENET. Waldmann *et al* [35] reported that ENET usually detects more true and false positive associations. In our case, this may result in an increased probability of having significant associations by chance. In turn, this can lead to reduced power. On the other hand, ENET selected some genes that were not selected by LASSO, mainly due to the correlated structure of the parameters. An example of this is displayed in Fig 4, showing that *MMP7* has three correlation nets that probably are responsible for the gene selection with ENET and not with LASSO. These comparisons are shown in S4 Table.

Regarding the differences between the models, 13/25 and 6/20 significant genes in the CpG and 6/20 the SNP models, respectively, were not significant in the Global model. It is reported in the literature that 10% of SNPs are associated with gene expression and DNA methylation [36,37], hence DNA methylation may confound or modify the association between SNP and gene expression. Even though this is a potential explanation, discordances resulting from sample size cannot be discarded since the penalty function is selected by CV. However, k = 5 was used to apply the k-fold CV to decrease the problem of small sample size without increasing bias. In the reverse scenario, 16 genes were selected exclusively in the Global model. Some of the genes identified had high deviances and borderline p-values, probably because the Global models increase the deviance due to the addition of more information when integrating data. For the nonsignificant genes, the explanation could be the existence of an interaction effect between SNPs and CpGs (S5 Table). This further supports the importance of integrating *omics* data to discover hidden information.

The validity of the strategy that we have developed, and of the results obtained, is supported by the fact that 75% (36/48) of the genes identified in the discovery phase were replicated using TCGA data by applying the same strategy. This represents 64% of all gene models found since some of the genes overlap between models and approaches. Also, the null results of the simulation study indicate that the significant associations found are unlikely to be due to chance.

Importantly, several of the genes that emerged from our analyses have been previously shown to be important in bladder cancer biology, including *KRT20*, *IGF2*, *CTSE*, *ANXA10* and *CRH*. These genes have already been proposed for a panel of molecular markers to improve the diagnosis and follow-up of UBC as part of a 12-gene expression urine signature to identify patients suffering from UBC and predict tumor aggressiveness [38]. The five genes aforementioned were also replicated in the TCGA data. Furthermore, *KRT20*, *IGF2*, and *CTSE* have also been previously associated with UBC. *KRT20* is a highly specific marker of umbrella cells in normal urothelium and its expression is commonly altered in papillary non muscle-invasive UBC as well as in muscle-invasive UBC. It has been proposed that the correlation between *FGFR3* mutations with normal *KRT20* expression pattern may indicate that the mutation occurs earlier [39]. Loss of imprinting (LOI) is a common epigenetic event in cancer and a LOI of *IGF2* has been reported in UBC [40]. In our analysis, *IGF2* was detected in the SNP and CPG models suggesting that both type of factors may be involved in regulating the expression levels of this gene. *CTSE* expression was significantly associated with progression-free survival in pTa tumors in a study of gene expression profiles in UBC [41].

We also performed a gene enrichment analysis to assess whether the significant genes had related biological functions. The cluster with the highest ES was”Extracellular region, secreted, and signal peptide”. Secreted proteins are known to play a crucial role in cell signaling and the cellular secretome has a major impact on multiple aspects of tumor cell biology (cell growth, migration, invasion, and angiogenesis) [42]. One cluster highly enriched in keratins points to the regulation of cell differentiation, known to be important in the molecular classification of UBC. In addition, some genes—including *S100A9* and *S100A2—*were grouped under the “EF hand and calcium ion binding” term. The *S100* family is composed of, at least, 24 members carrying the Ca^2+^ binding EF-hand motif. Expression of *S100* protein family members is regulated during inflammation and carcinogenesis and has been associated with poor prognosis in patients with UBC [43]. Other studies have reported an overexpression of *S100A9* in UBC tissue [44,45].

Limitations of this work are the small sample size of the discovery phase study, due to the lack of enough fresh tumor tissue from the same set of individuals, and the lack of a comparable and independent UBC patient series with the 3-*omics* data available to replicate our results. While the discovery EPICURO study recruited all patients with UBC, the TCGA project focused on muscle-invasive UBC. In addition, different highthroughput technologies/platforms were used in each of the studies. The SNP arrays genotyped different SNPs and, consequently, provided different genomic coverage. The TCGA used a DNA methylation array of 450k with much higher resolution than the 27k the one used in the EPICURO study. Finally, the use of different technologies to measure transcriptomics is a considerable limitation. In the EPICURO discovery phase, gene expression levels were measured with microarrays which provide relative values at probe set level, that is, for one gene different expression levels can be obtained from each mapped probe, while in the TCGA study gene expression was measured with RNA-seq which gives absolute gene expression values. These differences between data sets introduce a massive heterogeneity that makes the replication even more difficult. In spite of that, we replicated 75% of the identified genes (64% of the models) with TCGA data, providing strong support to the appropriateness of our approach and the relevance of the results obtained. Another potential limitation is the fact that tumor samples are heterogeneous regarding neoplastic cell content and stromal cell composition. Consequently, we checked the expression of all significant genes in a panel of UBC cell lines with available microarray expression data [46] and found that all but one (*IGJ*) are expressed in urothelial tumor cells, indicating that our analyses likely reflect genomic regulatory events in the tumor cells. It is, however, likely that relevant genomic interactions control gene expression not only in neoplastic cells but also in the stroma. Given the importance of the latter in tumor progression, further integrative *omics* studies using microdissected material will be highly informative.

One important strength of the approach used here is the lack of need to filter by LD in SNPs, or grouping CpGs within CpG islands, when dealing with a huge number of heterogeneous and correlated parameters delivered by different arrays. This emanates from the fact that LASSO and ENET can deal with highly correlated variables while performing variable selection. By performing data reduction/filtering before applying the statistical methods, there is a chance to filter out the functional SNPs and/or CpGs and thereby lose their association with gene expression. The adaptation of a strategy that performs a permutation and the maxT algorithm to assess p-values and to correct by MT, avoiding a double permutation and therefore reducing computational time, is also worthwhile emphasizing. In this regard, the permutation-based method considers the permutation of individuals within each gene, allowing to control for the possible dependence structure between genes. In addition, the MaxT algorithm is a permutationbased FWER controlling procedure which is adapted to the correlation structure found in the data and has been shown to be asymptotically optimal under dependence [47]

In summary, we demonstrate that the integration of multiple *omics* data types allows the identification of hidden mechanisms that were missed when analyzing single *omics* data types individually. There is an urgent need to develop statistical methods to fill the gap between the huge amount of data generated and the mechanistic understanding of complex diseases. Here, we present two penalized regression methods (LASSO and ENET) in combination with a permutation–based strategy (permutation-based MaxT method) to deal with common problems found in integrative analysis: heterogeneity between data types, number of individuals much smaller than the parameters to assess, multicollinearity, and sparseness to facilitate the interpretation of the results. This approach is flexible and easy to implement in different *omics* data and diseases as well as when considering interaction terms in the model.

We contribute to the field with a methodological development and with several significant and sound molecular associations conforming part of the genetic architecture of UBC. By using this cancer as an example, we conclude that modeling the intricacy of *omics* data variation with appropriate statistical strategies will certainly improve our knowledge of the mechanisms involved in complex diseases.

### Data availability statement

Common genetic variation (GSE51641), DNA methylation (GSE71666), and gene expression (GSE71576) data for the discovery phase are available in GEO.

## Supporting Information

S1 Excel SNP-LASSO model. Information about SNPs associated with gene expression using LASSO with SNP model for the discovery and replication phase.
(XLSX)

S2 Excel CPG-LASSO model. Information about CpGs associated with gene expression using LASSO with CPG model for the discovery and replication phase.
(XLSX)

S3 Excel Global-LASSO model. Information about SNPs and CpGs associated with gene expression using LASSO with Global model for the discovery and replication phase.
(XLSX)

S4 Excel SNP-ENET model. Information about SNPs associated with gene expression using ENET with SNP model for the discovery and replication phase.
(XLSX)

S5 Excel CPG-ENET model. Information about CpGs associated with gene expression using ENET with CPG model for the discovery and replication phase.
(XLSX)

S6 Excel Global-ENET model. Information about SNPs and CpGs associated with gene expression using ENET with Global model for the discovery and replication phase.
(XLSX)

S1 Fig Deviance across the genome when applying LASSO for the SNP model for the simulated data. The number of genes simulated are 20,899 for 27 individuals using a multivariate normal distribution (μ = 8.4, σ^2^ = 0.4). No gene was significantly associated after the permutation-based MaxT algorithm.
(TIF)

S1 Table IDs corresponding to the 27 samples from the EPICURO data used in this analysis. (DOCX)

S2 Table IDs corresponding to the 238 samples from the TCGA data used in this analysis. (DOCX)

S3 Table Functional Annotation Clustering from DAVID tool (Enrichment score ≥ 1.3). (DOCX)

S4 Table Comparison of the deviance, p-value and SNPs and/or CpGs detected by each model between LASSO and ENET methods. (DOCX)

S5 Table Comparison of genes selected by each model (SNP, CpG and Global model) using LASSO. (DOCX)
