# Supplementary figures and images for: Integration Analysis of Three Omics Data Using Penalized Regression Methods: An Application to Bladder Cancer
Source: PLoS Genet. 2015 Dec 8;11(12):e1005689. doi: 10.1371/journal.pgen.1005689 (PMC4672920; doi:10.1371/journal.pgen.1005689)

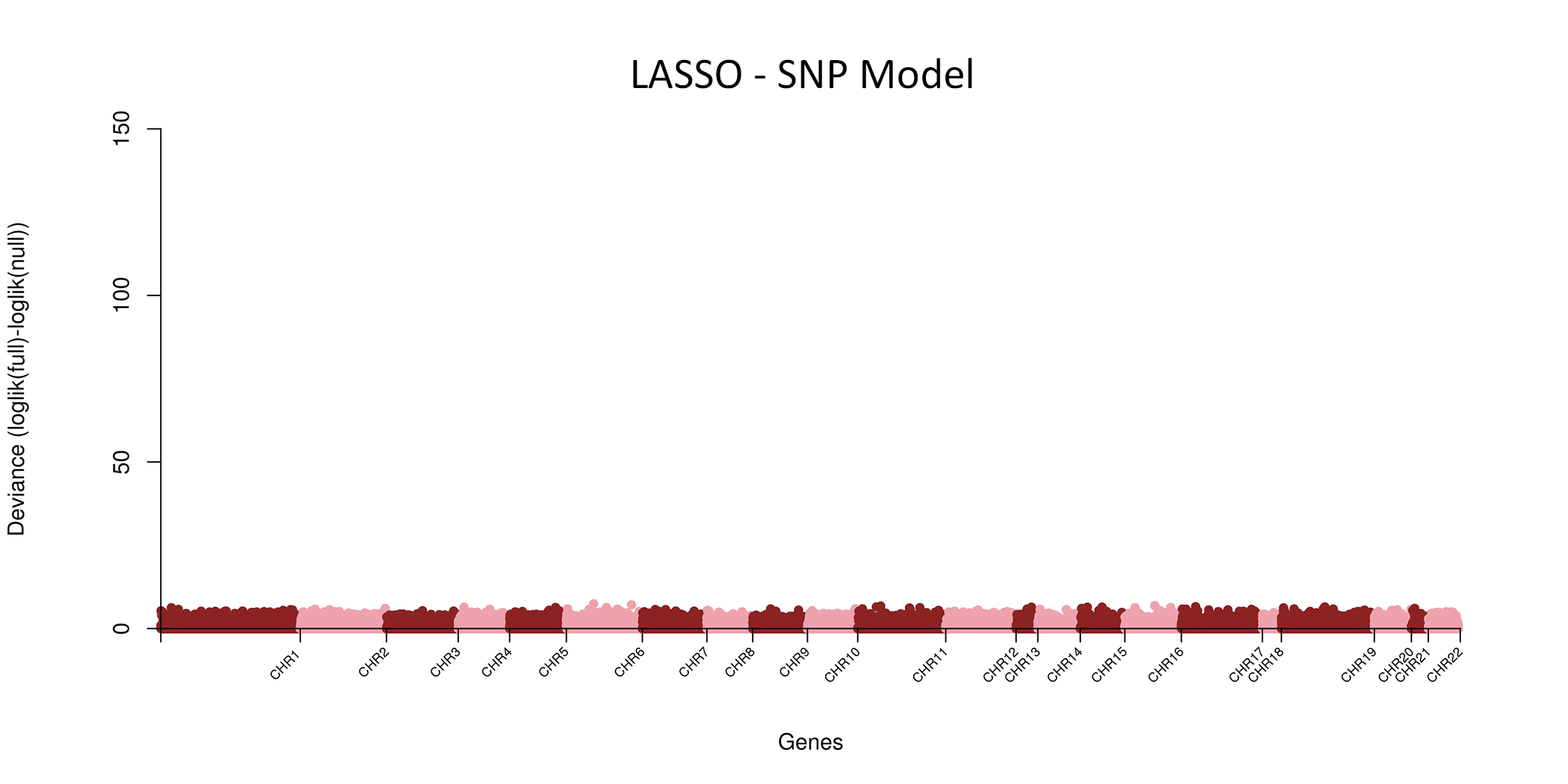

Supplement: S1 Fig — The number of genes simulated are 20,899 for 27 individuals using a multivariate normal distribution (μ = 8.4, σ2 = 0.4). No gene was significantly associated after the permutation-based MaxT algorithm. (TIF) [file pgen.1005689.s007.tif]
