# Supplementary material for: Integration Analysis of Three Omics Data Using Penalized Regression Methods: An Application to Bladder Cancer
Source: PLoS Genet. 2015 Dec 8;11(12):e1005689. doi: 10.1371/journal.pgen.1005689 (PMC4672920; doi:10.1371/journal.pgen.1005689)
Supplement: S1 Table — (DOCX) [file pgen.1005689.s008.docx]

| **GSE71666** | **GSE71576** | **GSE51641** |
| --- | --- | --- |
| 10090510 | 10090510 | 4118698416 |
| 10090910 | 10090910 | 4235966055 |
| 10091310 | 10091310 | 4235966030 |
| 10091710 | 10091710 | 4235966045 |
| 10091910 | 10091910 | 4118698575 |
| 10092810 | 10092810 | 4235966022 |
| 10093010 | 10093010 | 4239166109 |
| 10093110 | 10093110 | 4235966295 |
| 10093210 | 10093210 | 4118698560 |
| 10093310 | 10093310 | 4235966250 |
| 10093410 | 10093410 | 4118698403 |
| 10093510 | 10093510 | 4239166219 |
| 10093710 | 10093710 | 4118698451 |
| 10094010 | 10094010 | 4235966024 |
| 10094310 | 10094310 | 4239166062 |
| 10094410 | 10094410 | 4239166175 |
| 30105412 | 30105412 | 4118698428 |
| 30105711 | 30105711 | 4118698441 |
| 30106516 | 30106516 | 4235966298 |
| 30106619 | 30106619 | 4118698433 |
| 30107012 | 30107012 | 4235966076 |
| 30107610 | 30107610 | 4235966233 |
| 30107713 | 30107713 | 4235966253 |
| 30107919 | 30107919 | 4118698426 |
| 30108817 | 30108817 | 4235966029 |
| 30109911 | 30109911 | 4118698434 |
| 30110214 | 30110214 | 4118698427 |

**S1 Table: IDs corresponding to the 27 samples from EPICURO data used in this analysis**
