# Supplementary material for: Integration Analysis of Three Omics Data Using Penalized Regression Methods: An Application to Bladder Cancer
Source: PLoS Genet. 2015 Dec 8;11(12):e1005689. doi: 10.1371/journal.pgen.1005689 (PMC4672920; doi:10.1371/journal.pgen.1005689)
Supplement: S2 Table — (DOCX) [file pgen.1005689.s009.docx]

| A0S7 | A0YX | A0F0 | A0F6 | A0YN | A0YR | A0YO | A1HR |
| --- | --- | --- | --- | --- | --- | --- | --- |
| A1A3 | A20J | A20N | A20O | A20P | A20Q | A20T | A20U |
| A0C8 | A13J | A1A5 | A1A6 | A1A7 | A1AA | A1AB | A1AC |
| A1AG | A1AF | A20R | A20X | A2LA | A2LB | A27C | A2HX |
| A2I6 | A2PC | A3B3 | A3B4 | A3EE | A2EC | A2EF | A2EJ |
| A2EO | A2ES | A2C5 | A2HO | A2HQ | A2OE | A3JX | A3JW |
| A2LD | A3B6 | A3IT | A3IL | A3IN | A3B8 | A3MF | A3MI |
| A3B7 | A3MH | A3IB | A3IU | A3IS | A3IM | A2I4 | A3IV |
| A3B5 | A3JM | A3JZ | A3N6 | A3KJ | A3PH | A3PJ | A3PK |
| A3OQ | A3OS | A3JV | A3QG | A3QH | A3QI | A3QU | A3YL |
| A3X1 | A3X2 | A3X6 | A3Y1 | A3SJ | A3SL | A3SM | A3SN |
| A3SQ | A3SR | A3SS | A3VY | A3BM | A3OO | A3RC | A3RD |
| A3WS | A3WV | A0F1 | A0F7 | A0EZ | A42C | A3WW | A3ZE |
| A42R | A40E | A40G | A3Z7 | A42F | A42E | A47T | A47S |
| A47X | A47Y | A43N | A43P | A43S | A43U | A43X | A42P |
| A5UA | A5W6 | A5KE | A5KF | A5BY | A5BZ | A5C0 | A5C1 |
| A5RJ | A5Z6 | A4IJ | A4XJ | A541 | A43Y | A5BR | A5BS |
| A5BV | A5BX | A3Z9 | A4ZW | A2OF | A5ND | A4AC | A54R |
| A6AV | A6AW | A6B0 | A6B1 | A6B2 | A6B5 | A6B6 | A4TZ |
| A677 | A678 | A62N | A62O | A62P | A62S | A61P | A6I1 |
| A5RH | A6FZ | A6MB | A66R | A6FI | A6FN | A69X | A6DX |
| A6MF | A7DU | A6TF | A6TG | A6TH | A6TI | A76B | A763 |
| A72E | A7DV | A6TA | A6TB | A6TC | A6TD | A6TE | A6TK |
| A41N | A41P | A41Q | A41S | A78K | A78L | A78N | A78O |
| A20V | A1AE | A2I2 | A2EL | A3MG | A3NA | A3N5 | A3OP |
| A3SP | A3QF | A3YS | A47W | A5U8 | A5RI | A5BU | A5ZZ |
| A6C6 | A6ME | A767 | A6ZA | A13I | A20W | A1AD | A2I1 |
| A2EK | A3I6 | A3IE | A3IK | A3RB | A3WX | A3SO | A3WC |
| A3ZF | A47V | A42Q | A5NE | A5BT | A51V | A4U1 | A6I3 |
| A3IQ | A766 | A762 | A1HS | A3WY | A519 |  |  |

**S2 Table: IDs corresponding to the 238 samples from the TCGA data used in this analysis**
