# Supplementary material for: Integration Analysis of Three Omics Data Using Penalized Regression Methods: An Application to Bladder Cancer
Source: PLoS Genet. 2015 Dec 8;11(12):e1005689. doi: 10.1371/journal.pgen.1005689 (PMC4672920; doi:10.1371/journal.pgen.1005689)
Supplement: S3 Table — (DOCX) [file pgen.1005689.s010.docx]

**S3 Table: Functional Annotation Clustering from DAVID tool (Enrichment score ≥ 1.3)**

| **Cluster 1** | **Enrichment Score: 3.5** | | |  |  |
| --- | --- | --- | --- | --- | --- |
| Category | Term | Count | PValue | Genes | Benjamini |
| GOTERM_CC_FAT | GO:0005576~extracellular region | 17 | 1.05E-05 | OLFM4, CRTAC1, MSMB, IGJ, MMP7, IGF2, PIGR, TCN1, CXCL17, FREM2, SAA1, REN, IGHD, CRH, PLA2G2A, PTN, CP | 7.79E-04 |
| SP_PIR_KEYWORDS | Secreted | 15 | 1.41E-05 | OLFM4, CRTAC1, MSMB, S100A9, MMP7, IGF2, PIGR, TCN1, CXCL17, SAA1, REN, IGHD, CRH, PTN, CP | 2.02E-03 |
| SP_PIR_KEYWORDS | signal | 20 | 3.81E-05 | OLFM4, CRTAC1, MSMB, IGJ, MMP7, IGF2, PIGR, TCN1, CXCL17, SAA1, FREM2, REN, CRH, CTSE, CEACAM7, PLA2G2A, PTN, CEACAM6, CEACAM5, CP | 2.72E-03 |
| UP_SEQ_FEATURE | signal peptide | 20 | 4.17E-05 | OLFM4, CRTAC1, MSMB, IGJ, MMP7, IGF2, PIGR, TCN1, CXCL17, SAA1, FREM2, REN, CRH, CTSE, CEACAM7, PLA2G2A, PTN, CEACAM6, CEACAM5, CP | 8.55E-03 |
| GOTERM_CC_FAT | GO:0044421~extracellular region part | 11 | 1.02E-04 | OLFM4, CRTAC1, SAA1, FREM2, MSMB, REN, MMP7, PLA2G2A, PTN, IGF2, CP | 3.78E-03 |
| GOTERM_CC_FAT | GO:0005615~extracellular space | 9 | 2.74E-04 | OLFM4, SAA1, MSMB, REN, MMP7, PLA2G2A, PTN, IGF2, CP | 6.74E-03 |
| UP_SEQ_FEATURE | disulfide bond | 16 | 1.08E-03 | OLFM4, CRTAC1, MSMB, IGJ, IGF2, PIGR, TCN1, CXCL17, REN, IGHD, CTSE, CEACAM7, PLA2G2A, PTN, CEACAM6, CP | 1.05E-01 |
| SP_PIR_KEYWORDS | disulfide bond | 16 | 1.48E-03 | OLFM4, CRTAC1, MSMB, IGJ, IGF2, PIGR, TCN1, CXCL17, REN, IGHD, CTSE, CEACAM7, PLA2G2A, PTN, CEACAM6, CP | 6.81E-02 |
| SP_PIR_KEYWORDS | glycoprotein | 19 | 4.63E-03 | SLC38A4, OLFM4, CRTAC1, IGJ, KRT13, IGF2, TSPAN8, PIGR, TCN1, FREM2, REN, IGHD, CTSE, CEACAM7, CEACAM6, CEACAM5, SERPINB4, SERPINB3, CP | 1.24E-01 |
| UP_SEQ_FEATURE | glycosylation site:N-linked (GlcNAc...) | 14 | 1.37E-01 | SLC38A4, OLFM4, IGJ, TSPAN8, PIGR, TCN1, FREM2, REN, IGHD, CTSE, CEACAM7, CEACAM6, CEACAM5, CP | 9.20E-01 |
| **Cluster 2** | **Enrichment Score: 1.8** | | |  |  |
| Category | Term | Count | PValue | Genes | Benjamini |
| GOTERM_BP_FAT | GO:0032101~regulation of response to external stimulus | 4 | 5.63E-03 | SAA1, PLA2G2A, TSPAN8, IGF2 | 7.77E-01 |
| GOTERM_BP_FAT | GO:0050727~regulation of inflammatory response | 3 | 1.30E-02 | SAA1, PLA2G2A, IGF2 | 7.52E-01 |
| GOTERM_BP_FAT | GO:0006952~defense response | 5 | 5.03E-02 | SAA1, S100A9, CRH, PLA2G2A, IGF2 | 8.40E-01 |
| **Cluster 3** | **Enrichment Score: 1.7** | | |  |  |
| Category | Term | Count | PValue | Genes | Benjamini |
| GOTERM_BP_FAT | GO:0050708~regulation of protein secretion | 3 | 7.76E-03 | SAA1, IGF2, KRT20 | 7.49E-01 |
| GOTERM_BP_FAT | GO:0051046~regulation of secretion | 4 | 1.08E-02 | SAA1, CRH, IGF2, KRT20 | 7.65E-01 |
| GOTERM_BP_FAT | GO:0060341~regulation of cellular localization | 4 | 1.87E-02 | SAA1, CRH, IGF2, KRT20 | 8.13E-01 |
| GOTERM_BP_FAT | GO:0051223~regulation of protein transport | 3 | 2.79E-02 | SAA1, IGF2, KRT20 | 7.78E-01 |
| GOTERM_BP_FAT | GO:0070201~regulation of establishment of protein localization | 3 | 3.12E-02 | SAA1, IGF2, KRT20 | 7.84E-01 |
| GOTERM_BP_FAT | GO:0032880~regulation of protein localization | 3 | 3.96E-02 | SAA1, IGF2, KRT20 | 8.09E-01 |
| **Cluster 4** | **Enrichment Score: 1.5** | | | |  |
| Category | Term | Count | PValue | Genes | Benjamini |
| GOTERM_BP_FAT | GO:0007610~behavior | 6 | 3.95E-03 | CXCL17, SAA1, REN, S100A9, CRH, PTN | 8.78E-01 |
| GOTERM_BP_FAT | GO:0006935~chemotaxis | 3 | 5.17E-02 | CXCL17, SAA1, S100A9 | 8.29E-01 |
| GOTERM_BP_FAT | GO:0042330~taxis | 3 | 5.17E-02 | CXCL17, SAA1, S100A9 | 8.29E-01 |
| GOTERM_BP_FAT | GO:0007626~locomotory behavior | 3 | 1.30E-01 | CXCL17, SAA1, S100A9 | 9.48E-01 |
| **Cluster 5** | **Enrichment Score: 1.4** | | | |  |
| Category | Term | Count | PValue | Genes | Benjamini |
| UP_SEQ_FEATURE | region of interest:Coil 2 | 3 | 9.54E-03 | KRT5, KRT13, KRT20 | 4.82E-01 |
| UP_SEQ_FEATURE | region of interest:Linker 12 | 3 | 9.54E-03 | KRT5, KRT13, KRT20 | 4.82E-01 |
| UP_SEQ_FEATURE | region of interest:Coil 1B | 3 | 1.13E-02 | KRT5, KRT13, KRT20 | 4.44E-01 |
| UP_SEQ_FEATURE | region of interest:Coil 1A | 3 | 1.13E-02 | KRT5, KRT13, KRT20 | 4.44E-01 |
| UP_SEQ_FEATURE | region of interest:Linker 1 | 3 | 1.13E-02 | KRT5, KRT13, KRT20 | 4.44E-01 |
| UP_SEQ_FEATURE | region of interest:Rod | 3 | 1.16E-02 | KRT5, KRT13, KRT20 | 3.83E-01 |
| UP_SEQ_FEATURE | region of interest:Head | 3 | 1.23E-02 | KRT5, KRT13, KRT20 | 3.46E-01 |
| UP_SEQ_FEATURE | region of interest:Tail | 3 | 1.29E-02 | KRT5, KRT13, KRT20 | 3.18E-01 |
| SP_PIR_KEYWORDS | Intermediate filament | 3 | 1.38E-02 | KRT5, KRT13, KRT20 | 2.46E-01 |
| INTERPRO | IPR018039:Intermediate filament protein, conserved site | 3 | 1.42E-02 | KRT5, KRT13, KRT20 | 2.85E-01 |
| INTERPRO | IPR016044:Filament | 3 | 1.42E-02 | KRT5, KRT13, KRT20 | 2.85E-01 |
| INTERPRO | IPR001664:Intermediate filament protein | 3 | 1.46E-02 | KRT5, KRT13, KRT20 | 2.41E-01 |
| PIR_SUPERFAMILY | PIRSF002282:cytoskeletal keratin | 3 | 1.78E-02 | KRT5, KRT13, KRT20 | 3.84E-01 |
| SP_PIR_KEYWORDS | keratin | 3 | 4.29E-02 | KRT5, KRT13, KRT20 | 4.66E-01 |
| GOTERM_CC_FAT | GO:0005882~intermediate filament | 3 | 8.06E-02 | KRT5, KRT13, KRT20 | 7.12E-01 |
| GOTERM_CC_FAT | GO:0045111~intermediate filament cytoskeleton | 3 | 8.37E-02 | KRT5, KRT13, KRT20 | 6.60E-01 |
| GOTERM_MF_FAT | GO:0005198~structural molecule activity | 4 | 1.79E-01 | KRT5, MYBPC1, KRT13, KRT20 | 9.94E-01 |
| GOTERM_CC_FAT | GO:0044430~cytoskeletal part | 5 | 2.29E-01 | TNNT3, KRT5, MYBPC1, KRT13, KRT20 | 8.82E-01 |
| GOTERM_CC_FAT | GO:0005856~cytoskeleton | 5 | 4.84E-01 | TNNT3, KRT5, MYBPC1, KRT13, KRT20 | 9.93E-01 |
| SP_PIR_KEYWORDS | coiled coil | 5 | 6.92E-01 | OLFM4, KRT5, TRIM31, KRT13, KRT20 | 9.99E-01 |
| GOTERM_CC_FAT | GO:0043232~intracellular non-membrane-bounded organelle | 6 | 8.29E-01 | TNNT3, KRT5, MYBPC1, S100A9, KRT13, KRT20 | 1.00E+00 |
| GOTERM_CC_FAT | GO:0043228~non-membrane-bounded organelle | 6 | 8.29E-01 | TNNT3, KRT5, MYBPC1, S100A9, KRT13, KRT20 | 1.00E+00 |
| **Cluster 6** | **Enrichment Score: 1.3** | | |  |  |
| Category | Term | Count | PValue | Genes | Benjamini |
| GOTERM_BP_FAT | GO:0051046~regulation of secretion | 4 | 1.08E-02 | SAA1, CRH, IGF2, KRT20 | 7.65E-01 |
| GOTERM_BP_FAT | GO:0060341~regulation of cellular localization | 4 | 1.87E-02 | SAA1, CRH, IGF2, KRT20 | 8.13E-01 |
| GOTERM_BP_FAT | GO:0048585~negative regulation of response to stimulus | 3 | 2.19E-02 | SAA1, CRH, IGF2 | 8.14E-01 |
| GOTERM_BP_FAT | GO:0051047~positive regulation of secretion | 3 | 2.57E-02 | SAA1, CRH, IGF2 | 7.85E-01 |
| GOTERM_BP_FAT | GO:0006954~inflammatory response | 4 | 3.76E-02 | SAA1, S100A9, CRH, IGF2 | 8.17E-01 |
| GOTERM_BP_FAT | GO:0006952~defense response | 5 | 5.03E-02 | SAA1, S100A9, CRH, PLA2G2A, IGF2 | 8.40E-01 |
| GOTERM_BP_FAT | GO:0051050~positive regulation of transport | 3 | 9.21E-02 | SAA1, CRH, IGF2 | 9.24E-01 |
| GOTERM_BP_FAT | GO:0051240~positive regulation of multicellular organismal process | 3 | 1.07E-01 | SAA1, CRH, IGF2 | 9.27E-01 |
| GOTERM_BP_FAT | GO:0009611~response to wounding | 4 | 1.20E-01 | SAA1, S100A9, CRH, IGF2 | 9.41E-01 |
| GOTERM_BP_FAT | GO:0007267~cell-cell signaling | 4 | 1.57E-01 | S100A9, CRH, CEACAM6, IGF2 | 9.61E-01 |
| **Cluster 7** | **Enrichment Score: 1.3** | | | |  |
| Category | Term | Count | PValue | Genes | Benjamini |
| GOTERM_MF_FAT | GO:0005509~calcium ion binding | 8 | 4.19E-03 | CAPNS2, ANXA10, CRTAC1, FREM2, S100A9, MMP7, PLA2G2A, S100A2 | 3.57E-01 |
| SP_PIR_KEYWORDS | calcium | 6 | 3.55E-02 | CAPNS2, FREM2, S100A9, MMP7, PLA2G2A, S100A2 | 4.37E-01 |
| UP_SEQ_FEATURE | domain:EF-hand 1 | 3 | 6.58E-02 | CAPNS2, S100A9, S100A2 | 7.89E-01 |
| UP_SEQ_FEATURE | domain:EF-hand 2 | 3 | 6.58E-02 | CAPNS2, S100A9, S100A2 | 7.89E-01 |
| INTERPRO | IPR018249:EF-HAND 2 | 3 | 1.02E-01 | CAPNS2, S100A9, S100A2 | 6.75E-01 |
| INTERPRO | IPR018247:EF-HAND 1 | 3 | 1.04E-01 | CAPNS2, S100A9, S100A2 | 6.43E-01 |
| INTERPRO | IPR011992:EF-Hand type | 3 | 1.19E-01 | CAPNS2, S100A9, S100A2 | 6.62E-01 |
