# Supplementary material for: Integration Analysis of Three Omics Data Using Penalized Regression Methods: An Application to Bladder Cancer
Source: PLoS Genet. 2015 Dec 8;11(12):e1005689. doi: 10.1371/journal.pgen.1005689 (PMC4672920; doi:10.1371/journal.pgen.1005689)
Supplement: S4 Table — (DOCX) [file pgen.1005689.s011.docx]

**S4 Table: Comparison of the deviance, p-value and SNPs and/or CpGs detected by each model between LASSO and ENET methods**

|  |  | LASSO | | | ENET | | |
| --- | --- | --- | --- | --- | --- | --- | --- |
|  | GENE | Dev. | p.value | markers detected | Dev- | P.value | markers detected |
| SNP model | AIM2 | 55.8 | 0.1 | 6 | 91.8 | 0.13 | 18 |
|  | CRTAC1 | 66.2 | 0.03 | 18 | 72.8 | 0.24 | 23 |
|  | SCNN1A | 57.9 | 0.08 | 26 | 54.1 | 0.55 | 47 |
|  | CLIC6 | 75.3 | 0.01 | 30 | 75.3 | 0.17 | 104 |
|  | GSTT1 | 40.4 | 0.07 | 16 | 43.8 | 0.9 | 24 |
|  | ANXA10 | 0 | - | - | 137.0 | 0.01 | 17 |
|  | MSMB | 4.0 | 1 | 3 | 91.8 | 0.07 | 78 |
|  | MMP7 | 0 | - | - | 94.8 | 0.06 | 19 |
|  | TCN1 | 16.3 | 0.88 | 1 | 88.9 | 0.07 | 122 |
|  | IGF2 | 10.5 | 0.98 | 1 | 101.6 | 0.05 | 55 |
|  | GTSF1 | 50.4 | 0.23 | 2 | 109.6 | 0.05 | 19 |
|  | IGLJ3 | 0 | - | - | 97.7 | 0.05 | 182 |
| CPG model | S100A9 | 52.5 | 0.08 | 10 | 74.6 | 0.53 | 42 |
|  | S100A2 | 58.7 | 0.04 | 27 | 58.7 | 0.66 | 56 |
|  | CRTAC1 | 60.9 | 0.1 | 12 | 62.3 | 0.55 | 12 |
|  | SAA1 | 127.8 | 0.04 | 21 | 102.7 | 0.31 | 35 |
|  | MYBPC1 | 74.5 | 0.08 | 26 | 74.5 | 0.55 | 34 |
|  | SLC38A4 | 51.7 | 0.08 | 16 | 56.0 | 0.74 | 21 |
|  | GTSF1 | 46.7 | 0.1 | 3 | 82.2 | 0.28 | 9 |
|  | TSPAN8 | 67.2 | 0.05 | 9 | 69.3 | 0.55 | 9 |
|  | FREM2 | 70.2 | 0.06 | 16 | 76.0 | 0.52 | 27 |
|  | C15orf48 | 83.7 | 0.05 | 25 | 42.7 | 0.23 | 9 |
|  | CAPNS2 | 54.9 | 0.07 | 9 | 66.5 | 0.48 | 21 |
|  | KRT20 | 93.7 | <0.01 | 26 | 93.7 | 0.11 | 53 |
|  | SERPINB4 | 68.5 | 0.03 | 4 | 94.0 | 0.12 | 18 |
|  | CXCL17 | 46.8 | 0.1 | 8 | 45.5 | 0.24 | 7 |
|  | CLIC6 | 75.3 | 0.07 | 27 | 75.3 | 0.51 | 31 |
|  | TMEM45A | 57.3 | 0.08 | 13 | 66.2 | 0.48 | 61 |
|  | IGJ | 59.0 | 0.09 | 5 | 174.6 | 1 | 32 |
|  | UBD | 75.0 | 0.07 | 11 | 75.5 | 0.51 | 11 |
|  | ARHGEF35 | 49.6 | 0.09 | 9 | 51.9 | 0.8 | 14 |
|  | CRH | 56.7 | 0.1 | 4 | 60.1 | 0.59 | 5 |
|  | TRIM31 | 47.1 | 0.1 | 27 | 40.6 | 0.32 | 55 |
|  | MMP7 | 0 | - | - | 99.4 | 0.08 | 64 |
| Global model | S100A9 | 53.66 | 0.03 | 11 | 46.06 | 0.59 | 8 |
|  | CTSE | 60.7 | 0.06 | 12 | 70.12 | 0.23 | 17 |
|  | PLA2G2A | 71.4 | 0.01 | 10 | 66.78 | 0.26 | 32 |
|  | HMGCS2 | 53.3 | 0.02 | 8 | 58.21 | 0.18 | 10 |
|  | AIM2 | 61.5 | 0.06 | 8 | 104.88 | 0.12 | 24 |
|  | PIGR | 75.8 | <0.01 | 21 | 75.48 | 0.12 | 21 |
|  | TNNT3 | 44.9 | 0.09 | 26 | 36.19 | 0.82 | 59 |
|  | SCNN1A | 58.8 | 0.03 | 29 | 58.76 | 0.18 | 31 |
|  | KRT5 | 58.2 | 0.02 | 25 | 58.14 | 0.18 | 31 |
|  | FREM2 | 46.0 | 0.08 | 2 | 48.24 | 0.45 | 2 |
|  | OLFM4 | 60.0 | 0.06 | 10 | 61.90 | 0.16 | 11 |
|  | C15orf48 | 49.9 | 0.02 | 7 | 48.80 | 0.41 | 6 |
|  | KRT20 | 48.4 | 0.05 | 3 | 39.50 | 0.74 | 1 |
|  | KRT13 | 53.6 | 0.02 | 8 | 54.01 | 0.25 | 8 |
|  | CEACAM7 | 76.0 | <0.01 | 19 | 77.40 | 0.16 | 32 |
|  | CLIC6 | 45.1 | 0.09 | 4 | 47.35 | 0.50 | 6 |
|  | CP | 51.1 | 0.02 | 3 | 47.98 | 0.47 | 3 |
|  | IGJ | 58.4 | 0.03 | 2 | 94.72 | 0.17 | 16 |
|  | CRABP2 | 9.78 | 0.99 | 2 | 65.2 | 0.09 | 26 |
|  | REN | 0 | - | 0 | 84.3 | 0.03 | 22 |
|  | IGF2 | 89.01 | 0.17 | 11 | 92.1 | 0.04 | 15 |
|  | CEACAM5 | 90.85 | 0.19 | 22 | 92.1 | 0.06 | 26 |
|  | NLRP2 | 14.26 | 0.93 | 2 | 84.2 | 0.04 | 34 |
