# Supplementary material for: Integration Analysis of Three Omics Data Using Penalized Regression Methods: An Application to Bladder Cancer
Source: PLoS Genet. 2015 Dec 8;11(12):e1005689. doi: 10.1371/journal.pgen.1005689 (PMC4672920; doi:10.1371/journal.pgen.1005689)
Supplement: S5 Table — (DOCX) [file pgen.1005689.s012.docx]

**S5 Table: Comparison of genes selected by each model (SNP, CpG and Global model) using LASSO.**

|  | Global model | | | | SNP model | | | CPG model | | |
| --- | --- | --- | --- | --- | --- | --- | --- | --- | --- | --- |
| GENE | Dev | P | SNPs | CPGs | Dev | P | SNPs (common) | Dev | P | CPGs (common) |
| *GSTM1* | 79.9 | 0.03 | 12 | 5 | 0.0 | - | 0 | 0.0 | - | 0 |
| *TMEM45A* | 57.3 | 0.01 | 12 | 1 | 53.1 | 0.23 | 14 (11) | 1.2 | 1.0 | 1 (1) |
| *ANXA10* | 153.3 | 0.01 | 22 | 0 | 0.0 | - | 0 | 0.0 | - | 0 |
| *ALDH7A1* | 41.1 | 0.05 | 6 | 0 | 41.1 | 0.36 | 6 (6) | 6.2 | 1.0 | 1 (0) |
| *UBD* | 53.7 | 0.04 | 2 | 4 | 3.1 | 1.0 | 1 (1) | 91.0 | 0.22 | 13 (4) |
| *PTN* | 81.6 | 0.04 | 14 | 0 | 79.3 | 0.04 | 12 (12) | 0.0 | - | 0 |
| *IGF2* | 77.95 | 0.02 | 8 | 2 | 31.3 | 0.21 | 4 (3) | 92.3 | 0.23 | 13 (2) |
| *SLC38A4* | 57.8 | 0.01 | 18 | 2 | 0.0 | - | 0 | 13.3 | 0.94 | 3 (2) |
| *SERPINB4* | 78.0 | 0.02 | 6 | 0 | 91.7 | <0.01 | 13 (6) | 17.0 | 0.90 | 1 (0) |
| *SERPINB3* | 142.3 | 0 | 1 | 0 | 171.6 | 0.80 | 29 (11) | 25.2 | 0.46 | 1 (0) |
| *CEACAM5* | 88.9 | 0.02 | 13 | 5 | 0.0 | - | 0 (0) | 77.6 | 0.05 | 16 (5) |
| *AIM2* | 10.7 | 0.95 | 1 (1) | 0 | 107.5 | 0.02 | 24 |  |  |  |
| *FCGR3A* | 54.5 | 0.55 | 23 (14) | 4 | 45.1 | 0.04 | 24 |  |  |  |
| *AGMO* | 51.4 | 0.06 | 18 (18) | 0 | 45.2 | 0.04 | 13 |  |  |  |
| *PTN* | 81.6 | 0.04 | 14 (11) | 0 | 79.3 | 0.04 | 12 |  |  |  |
| *ARHGEF35* | 48.4 | 0.10 | 7 (5) | 1 | 40.5 | 0.05 | 5 |  |  |  |
| *SAA2* | 16.8 | 0.72 | 5 (3) | 1 | 69.3 | 0.04 | 13 |  |  |  |
| *IGHD* | 66.9 | 0.11 | 9 (9) | 2 | 71.7 | 0.01 | 10 |  |  |  |
| *SERPINB4* | 78.0 | 0.02 | 6 (6) | 0 | 91.7 | 0 | 13 |  |  |  |
| *CEACAM6* | 14.7 | 1.0 | 0 | 1 | 70.4 | 0.02 | 9 |  |  |  |
| *CLIC6* | 75.3 | 0.09 | 25 (14) | 2 | 73.0 | 0.02 | 21 |  |  |  |
| *PLA2G2A* | 88.6 | 0.77 | 24 | 6 (6) |  |  |  | 72.9 | 0.04 | 12 |
| *HMGCS2* | 0.0 | - | 0 | 0 |  |  |  | 58.7 | 0.04 | 10 |
| *S100A8* | 63.1 | 0.10 | 3 | 4 (3) |  |  |  | 55.1 | 0.04 | 4 |
| *AIM2* | 10.7 | 0.95 | 1 | 0 |  |  |  | 70.3 | 0.04 | 10 |
| *PIGR* | 0.0 | - | 0 | 0 |  |  |  | 65.0 | 0.04 | 9 |
| *IGJ* | 59.0 | 0.16 | 3 | 2 (2) |  |  |  | 70.8 | 0.05 | 4 |
| *BHMT* | 0 | - | 0 | 0 |  |  |  | 49.4 | 0.05 | 9 |
| *LCN2* | 70.7 | 0.08 | 11 | 6 (5) |  |  |  | 49.7 | 0.05 | 6 |
| *MSMB* | 0.0 | - | 0 | 0 |  |  |  | 77.3 | 0.02 | 8 |
| *TCN1* | 21.7 | 0.87 | 1 | 1 (1) |  |  |  | 55.1 | 0.04 | 8 |
| *KRT5* | 12.3 | 0.98 | 0 | 1 (1) |  |  |  | 58.2 | 0.04 | 25 |
| *CAPNS2* | 62.7 | 0.08 | 15 | 1 (1) |  |  |  | 50.7 | 0.04 | 7 |
| *KRT13* | 63.3 | 0.09 | 8 | 4 (4) |  |  |  | 52.3 | 0.04 | 7 |
| *C3* | 42.1 | 0.19 | 10 | 4 (3) |  |  |  | 629 | 0.04 | 21 |
| *CEACAM7* | 44.7 | 0.28 | 2 | 3 (2) |  |  |  | 77.5 | 0.02 | 25 |
| *CEACAM5* | 88.9 | 0.02 | 13 | 5 (5) |  |  |  | 77.6 | 0.05 | 16 |
| *NLRP2* | 0.0 | - | 0 | 0 |  |  |  | 73.1 | 0.05 | 16 |
